# Predicting ecosystem state changes in shallow lakes using an aquatic ecosystem model: Lake Hinge, Denmark, an example

**DOI:** 10.1002/eap.2160

**Published:** 2020-06-11

**Authors:** Tobias Kuhlmann Andersen, Anders Nielsen, Erik Jeppesen, Fenjuan Hu, Karsten Bolding, Zhengwen Liu, Martin Søndergaard, Liselotte S. Johansson, Dennis Trolle

**Affiliations:** ^1^ Department of Bioscience Aarhus University 8600 Silkeborg Denmark; ^2^ Sino‐Danish Center for Education and Research University of Chinese Academy of Sciences Beijing 100049 China; ^3^ State Key Laboratory of Lake Science and Environment Nanjing Institute of Geography and Limnology Chinese Academy of Sciences Nanjing 210008 China; ^4^ Department of Ecology Jinan University Guangzhou 510632 China

**Keywords:** aquatic ecosystem modeling, critical nutrient loads, FABM‐PCLake, General Ocean Turbulence Model, lake restoration, predictive ecology, regime shifts, shallow lakes, water quality

## Abstract

In recent years, considerable efforts have been made to restore turbid, phytoplankton‐dominated shallow lakes to a clear‐water state with high coverage of submerged macrophytes. Various dynamic lake models with simplified physical representations of vertical gradients, such as PCLake, have been used to predict external nutrient load thresholds for such nonlinear regime shifts. However, recent observational studies have questioned the concept of regime shifts by emphasizing that gradual changes are more common than sudden shifts. We investigated if regime shifts would be more gradual if the models account for depth‐dependent heterogeneity of the system by including the possibility of vertical gradients in the water column and sediment layers for the entire depth. Hence, bifurcation analysis was undertaken using the 1D hydrodynamic model GOTM, accounting for vertical gradients, coupled to the aquatic ecosystem model PCLake, which is implemented in the framework for aquatic biogeochemical modeling (FABM). First, the model was calibrated and validated against a comprehensive data set covering two consecutive 7‐yr periods from Lake Hinge, a shallow, eutrophic Danish lake. The autocalibration program Auto‐Calibration Python (ACPy) was applied to achieve a more comprehensive adjustment of model parameters. The model simulations showed excellent agreement with observed data for water temperature, total nitrogen, and nitrate and good agreement for ammonium, total phosphorus, phosphate, and chlorophyll *a* concentrations. Zooplankton and macrophyte coverage were adequately simulated for the purpose of this study, and in general the GOTM‐FABM‐PCLake model simulations performed well compared with other model studies. In contrast to previous model studies ignoring depth heterogeneity, our bifurcation analysis revealed that the spatial extent and depth limitation of macrophytes as well as phytoplankton chlorophyll‐*a* responded more gradually over time to a reduction in the external phosphorus load, albeit some hysteresis effects still appeared. In a management perspective, our study emphasizes the need to include depth heterogeneity in the model structure to more correctly determine at which external nutrient load a given lake changes ecosystem state to a clear‐water condition.

## Introduction

Lakes are known to be vulnerable to changes in climate and human pressures (Capon and Bruun 2015), and across the globe lake ecosystems are deteriorating with implications, not least for drinking water quality (Smith and Schindler 2009). Therefore, careful management of lakes is needed to ensure continuation of the efforts to restore their ecological integrity (Jeppesen et al. 2005*b*) to fulfill the UN Sustainable Development Goal 6 (“Ensure availability and sustainable management of water and sanitation for all”; UN 2018) and satisfy the requirements of the EU Water Framework Directive (annex V, 2000/60/EC). To this end, lake models support management decisions by providing lake managers with the necessary information to, for instance, quantify the effectiveness of measures (Hilt et al. 2017) to reach these goals.

In the literature on freshwater ecosystems and their management, the concepts of regime shifts (Scheffer and Carpenter 2003), alternative or multiple stable states (Scheffer et al. 1993), and critical transitions (Scheffer et al. 2009), hereafter referred to as “regime shifts,” have been used extensively. In general, the diverse terminology describes thresholds or tipping points between alternative stable states or regimes at which a sudden, nonlinear change in an ecosystem occurs due to incremental changes in pressure (Groffman et al. 2006). An outcome of the regime shift theory is the term “critical nutrient load” that describes the external nutrient load where a shallow lake will transition from a turbid, phytoplankton‐dominated state to a clear‐water, macrophyte‐dominated state following a nutrient load reduction or vice versa (Janssen et al. 2017).

Several kinds of mathematical models have been used to describe and elucidate the mechanisms behind shifts in states (Scheffer and Carpenter 2003). The zero‐dimensional lake ecosystem model PCLake (Janse 2005) is a process‐based model that has undergone continued development for two decades with the original aim to be able to capture regime shifts between turbid and clear‐water states in temperate shallow lakes (Janse 2005, Mooij et al. 2010). The PCLake model was shown to be capable of simulating regime shifts with hysteresis effects in a bifurcation analysis with PCLake applying a data set of 43 temperate shallow lakes (Janse 1997, Janse et al. 2008). The model has also been subject to both sensitivity analysis (Janse et al. 2010) and Bayesian calibration using a combined data set on total phosphorus (TP), chlorophyll *a* (chl *a*), macrophyte cover, and Secchi depth including more than 40 temperate shallow lakes (Aldenberg et al. 1995).

Recently, several studies and reviews have questioned the concept of regime shifts in lake ecosystems as only few studies have actually been able to detect a sudden, nonlinear shift in shallow lake ecosystems in their response to changes in nutrient load (Jeppesen et al. 2007, Capon et al. 2015, Spears et al. 2017). By way of example, a major gradual reduction of phytoplankton chl *a* concentrations was observed for several shallow Danish lakes following decreased TP concentrations (Jeppesen et al. 2007) and a gradual rather than a sudden loss of macrophyte volume was apparent from a data set of 39 north temperate lakes undergoing eutrophication (Sayer et al. 2010*b*). Overall, long‐term observations from north temperate lakes suggest that the two alternative stable states are less stable than originally anticipated as macrophytes loss or recovery seems more gradual (Jeppesen et al. 2007, Sayer et al. 2010*b*). Therefore, it is relevant also to question the models that support this concept, their underlying structure, and the assumption that models are able to predict a critical nutrient load that would lead to a change in state for a specific lake.

An important assumption in regime shift theory is that “vegetation disappears when a critical turbidity is exceeded” (Scheffer and Van Nes 2007), and this is implicit in the structure of the original, zero‐dimensional (0D) PCLake model where the lake is modeled as a fully mixed water column with one uniform water depth. By presenting the lake as a box of one depth, one ignores depth heterogeneity, here defined as possible occurrence of vertical gradients in, for instance, temperature and nutrient concentrations, and including a sediment layer in connection to each water layer. The 0D box‐model may be an important reason for the drastic simulated regime shift with a strong hysteretic effect found in the bifurcation analysis with PCLake. This has an “all or nothing” effect on several trophic levels, as pointed out by Mooij et al. (2010), and this was also indicated in a sensitivity analysis of the critical P load for regime shifts with varying uniform water depths (Janse et al. 2008). In contrast, theoretical examinations suggest that more accurate physical representations of ecosystems (e.g., a 1D water column model instead of a 0D box model) may render a more gradual ecosystem response to changes in external forcing (van Nes and Scheffer 2005).

In this study, we investigated if (1) model simulations of the (re)establishment of macrophytes would occur at higher external P load when accounting for depth heterogeneity (the possibility of vertical gradients and inclusion of a sediment layer in each water layer) due to the modeling of the more favorable light conditions at the top sediments allowing macrophytes to grow and (2) if simulated changes in ecosystem state would become more gradual when the models included depth heterogeneity due to, among other factors, the faster re‐establishment of macrophytes in the shallow littoral zone.

With the development of FABM‐PCLake (Hu et al. 2016), it has recently become possible to investigate the effects of depth heterogeneity in shallow lake modelling by coupling FABM‐PCLake with the one‐dimensional (1D) General Ocean Turbulence Model (GOTM, [Burchard et al. 1999]) into a single combined 1D model (GOTM‐FABM‐PCLake). In this study, we set up, calibrated, and validated GOTM‐FABM‐PCLake against a comprehensive data set on shallow, eutrophic Lake Hinge, Denmark, covering a 14‐yr period (two 7‐yr periods for calibration and validation, respectively). The newly developed auto‐calibration tool Auto‐Calibration Python (ACPy) was applied in a multi‐step calibration procedure with 140+ model parameters. During the simulation period, observations show that the lake recovered from past high nutrient loads and showed a slight increase in macrophyte coverage. Moreover, we undertook a bifurcation analysis based on the validated model to assess its responses in eutrophication and oligotrophication scenarios and to analyze the simulated ecosystem dynamics in the transitional phase between turbid and clear‐water states. We discussed and compared the results of the bifurcation analysis of the 1D GOTM‐FABM‐PCLake model with previously published 0D PCLake model studies and bifurcation analyses. As the new coupled GOTM‐FABM‐PCLake model proved able to simulate more realistic changes in macrophyte biomass and coverage to reduced nutrient load, we suggest that it may potentially also predict more realistic responses to a reduction of the P load than the currently available alternative models.

## Methods and Materials

### Study site and sampling

**Fig. 1:**
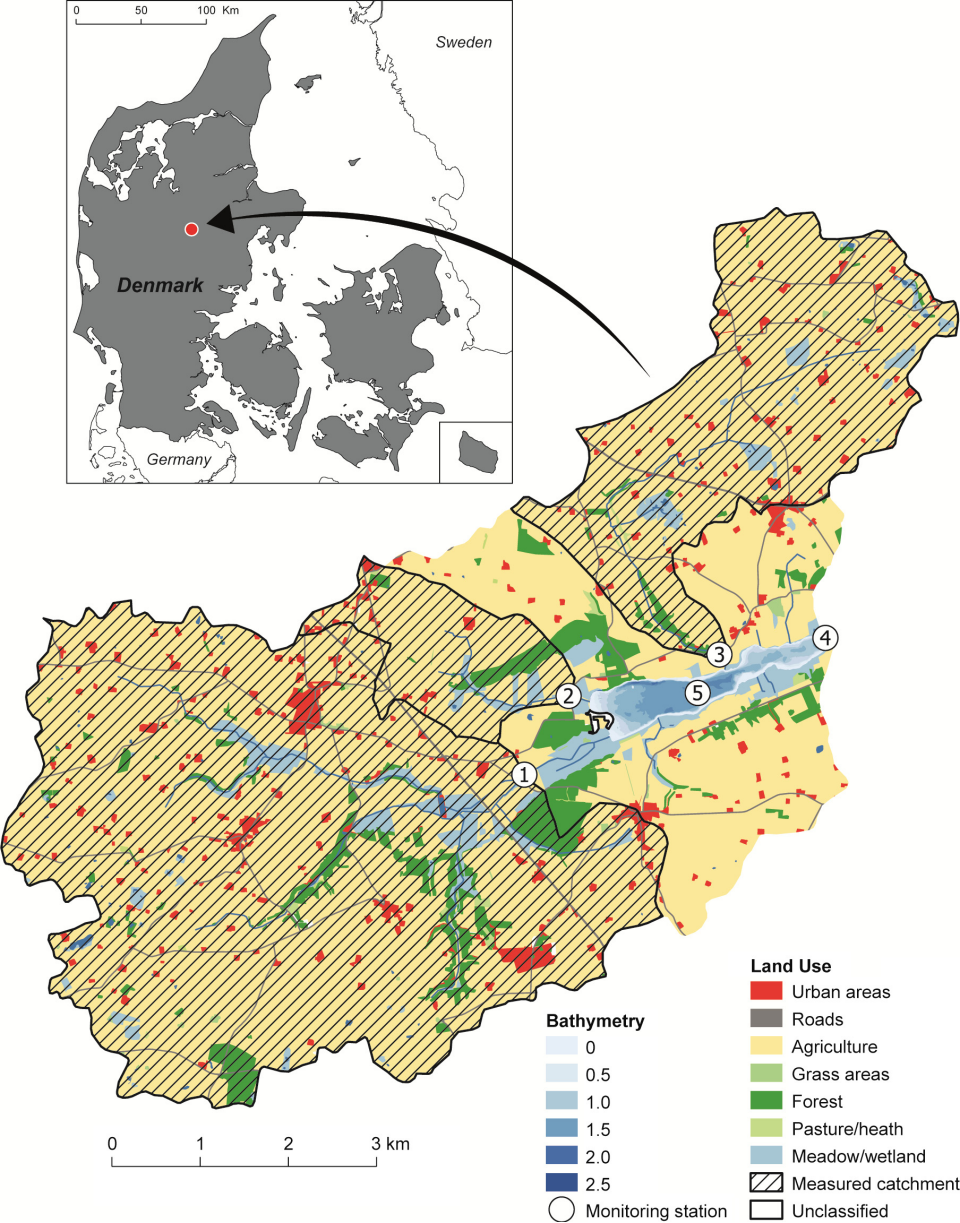
Study area covering the catchment of Lake Hinge, Denmark. Monitoring stations 1, 2, and 3 cover inlet streams from gauged catchment areas (shaded), station 4 represents the lake outlet and station 5 the in‐lake water quality sampling station. Land use classifications are derived from Nielsen et al. (2000).

Located in a moraine landscape in the central part of Jutland, Denmark, Lake Hinge is a small (0.91 km^2^), shallow lake with a mean depth of 1.2 m and a maximum depth of 2.6 m. The climate is temperate with an annual mean precipitation of 803 mm (1990–2011) and a mean winter (October–April) and summer (May–September) temperature of 3.7°C and 14.5°C, respectively (Riddersholm and Scharling 2010, Dee et al. 2011). The lake receives its water from precipitation and iron‐rich streams, mainly three inlets in the western and northern parts of the lake, and it has one outlet to the east. It has a short hydraulic residence time of 16 and 32 d in winter and summer, respectively. The near‐shore surroundings are relatively flat and consist mainly of pasture areas allowing for wind exposure. The catchment area (approximately 55 km^2^) comprises mainly agriculture (93%) with a small area of forest (5%) and urban areas (Fig. 1; Nielsen et al. 2000).

In 1989, Lake Hinge was included in the National Monitoring and Assessment Program for the Aquatic and Terrestrial Environment in Denmark (Kronvang et al. 1993) since it was considered representative of a substantial portion of Danish lakes affected by agricultural activity (Moeslund 1995). During 1990–2006, flow and nutrient (total nitrogen [TN], nitrate [NO_3_], ammonium [NH_4_], TP, and phosphate [PO_4_]) concentrations in the main inlets (monitoring station 1, 2, and 3 in Fig. 1), draining 77% of the catchment, were based on a times series of monthly averages according to the Danish national monitoring program (Thodsen et al. 2019) combined with estimates from the DK‐QNP model adapted to Danish catchments for the ungauged catchment area (Windolf et al. 2011). The particulate organic fractions were estimated as the difference between total and inorganic nutrient concentrations at all three inlets (estimated to 75% and 90% for organic P and N, respectively) and applied uniformly throughout the entire simulation period.

Lake water nutrient and phytoplankton chl *a* sampling was done once every winter month (except during ice cover) and twice each summer month. Samples for analysis of water quality parameters were taken at different depths to a maximum of twice the measured Secchi depth and then pooled according to the Danish national monitoring program (Johansson and Lauridsen 2017); the mean sampling depth usually ranging between 0.2 and 1.0 m. According to Danish standards (Order no 523 of 01/05/2019 historical), the maximum allowed relative measurement confidence limits for water nutrient and chl *a* analysis are 15% and 20%, respectively. The confidence limits were plotted as an error band around water quality samples to visualize uncertainty in observations. These uncertainty ranges were plotted as an error band around the water quality samples to visualize uncertainty in observations. Lake water temperature and dissolved oxygen were also measured once every winter month (except during ice cover) and twice each summer month usually at the surface, middle, and bottom of the lake. For most years in the simulation period, samples were taken for monthly phyto‐ and mesozooplankton taxa identification and biomass estimates from the surface down to a maximum depth of twice the Secchi depth or in the entire water column, respectively, and then pooled (Johansson 2011, Johansson and Lauridsen 2012). The mean depth of the pooled water quality and plankton samples represented the sampled depth for observations when comparing the observations with model simulations. Zooplankton biomass estimates were based on a zooplankton taxa specific length‐to‐biomass relation (Hansen et al. 1992, Johansson 2011). For each phytoplankton sample, phytoplankton was identified to genus or species level and taxa specific biomass was estimated (Johansson and Søndergaard 2012). To correspond with FABM‐PCLake phytoplankton structure, phytoplankton genus biomass estimates were aggregated into three phytoplankton model groups: diatoms, cyanobacteria, and other algae. For all years in the simulation period, submerged macrophyte coverage was monitored during maximum abundance in July or August (Søndergaard et al. 2005*b*). Total biomasses of planktivorous and piscivorous fish were determined for 1992, 1997, and 2002 and depth limits and coverage of macrophytes were established for 10 out of 14 simulation years.

In the model simulation period (1990–2006), the lake experienced a decrease in the yearly mean TN load, from approximately 139 to 100  Mg N/yr, and a change in yearly mean TP load from approximately 2.8 to 3.1  Mg P/yr for the periods 1990–1994 and 2000–2004, respectively. This was within the inter‐annual variation recorded for the entire simulation period (Bjerring et al. 2014). In comparison, the summer mean (May–September) TN concentrations were between 1.78–2.62 and 1.49–1.86 mg N/L and the TP concentrations was between 0.16–0.22 and 0.15–0.19 mg P/L for the same two periods. Lake Hinge’s sediment is iron rich with a weight ratio of Fe:TP between 30 and 45, which is high compared with most other Danish lakes (Søndergaard et al. 1996). Observed summer mean chl‐*a* concentrations and macrophyte coverage ranged between 100 to 160 µg/L and 0 to 2%, respectively, in the simulation period. In the last 2 yr of the period (2005 and 2006), the ecological state of the lake improved slightly with an increase in macrophyte coverage from 0–2% to 5% and with summer mean chl *a* concentrations of 61 and 141 µg chl *a*/L (Johansson et al. 2016).

During a typical year in the monitoring period, spring algae bloomed around April, the community being initially dominated by the centric diatom *Cyclotella* sp., followed in most years by an increase in the biomass and ratio of the pennate diatom *Aulacoseira* sp. later in spring. Concurrently, the biomass of cyclopoid zooplankton increased, mainly consisting of *Cyclops vicinus* with a peak around May followed by a decline, most likely caused by diapause or fish fry predation (Hansen and Jeppesen^1992^). With high food availability and less competition, cladoceran zooplankton increased in biomass around June, *Daphnia cucullata* being the dominant species, leading to a decrease in chl *a* concentrations compared with the spring peak. In most summers, the phytoplankton community was dominated by diatoms, while in late summer and fall (August–September), the phytoplankton remained dominated by diatoms or by filamentous cyanobacteria.

### Model description

**Fig. 2:**
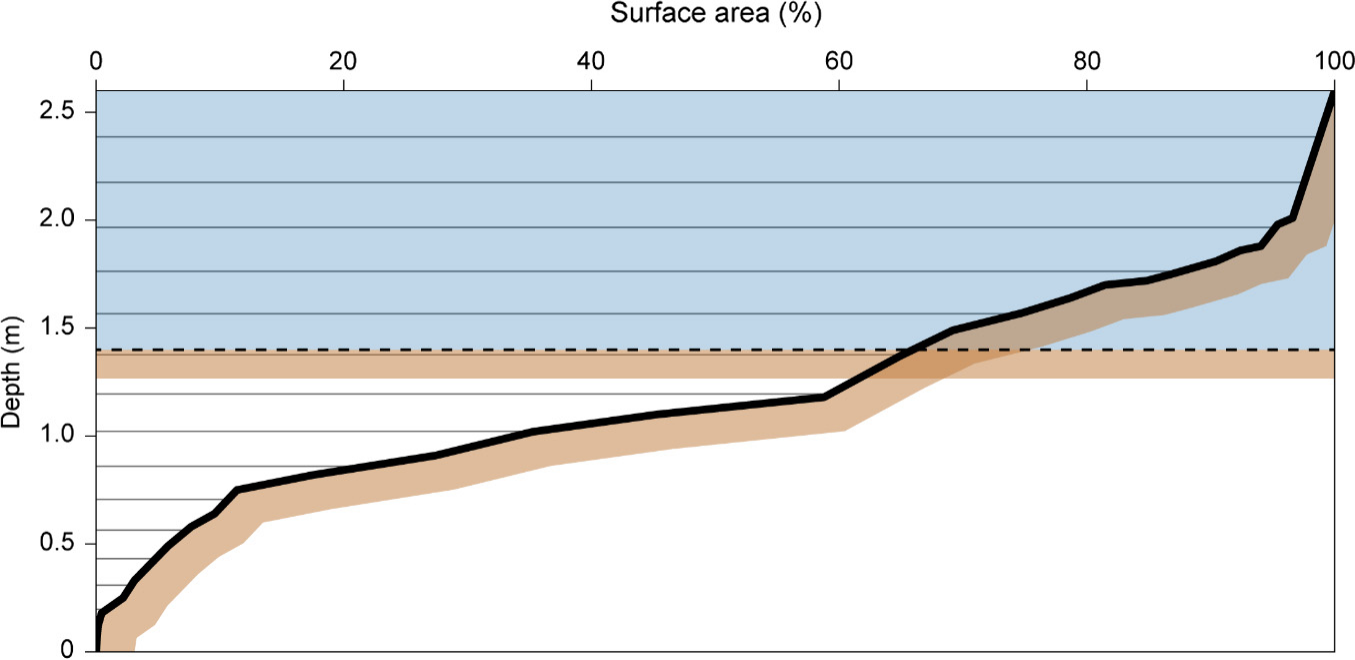
The relationship between lake level and surface area for Lake Hinge (thick black line) where level 0 m represents the deepest part of the lake. In the 1D GOTM‐FABM‐PCLake setup for Lake Hinge, the water column is composed of 16 water layers (gray lines) with a height between 10–21 cm and a sediment in each layer (illustrated by highlighted brown area). In comparison, the 0D PCLake setup for Lake Hinge (Janse et al. 2008) is a box model with one water layer (blue area) of 1.2 m (dashed line) with a connected sediment layer (illustrated by highlighted brown area).

This study applied the 1D water column model General Ocean Turbulence Model (GOTM) coupled with the lake ecosystem model FABM‐PCLake. The layer volumes and areas between layers and at the sediment‐water interface were derived from a lake‐specific hypsograph (i.e., the relation between depth and horizontal area) with max depth of 2.6 m and 16 depth levels in the water column with a grid zoom at the bottom (i.e., vertical grid sizes of 10–21 cm; Fig. 2). For further details on GOTM, see Burchard et al. (1999) and GOTM (2016). We utilized the k‐epsilon model for calculating the vertical mixing in the lake and the positive definite and conservative Extended Modified Patankar ordinary differential equation scheme for source and sink dynamics (Bruggeman et al. 2007).

**Fig. 3:**
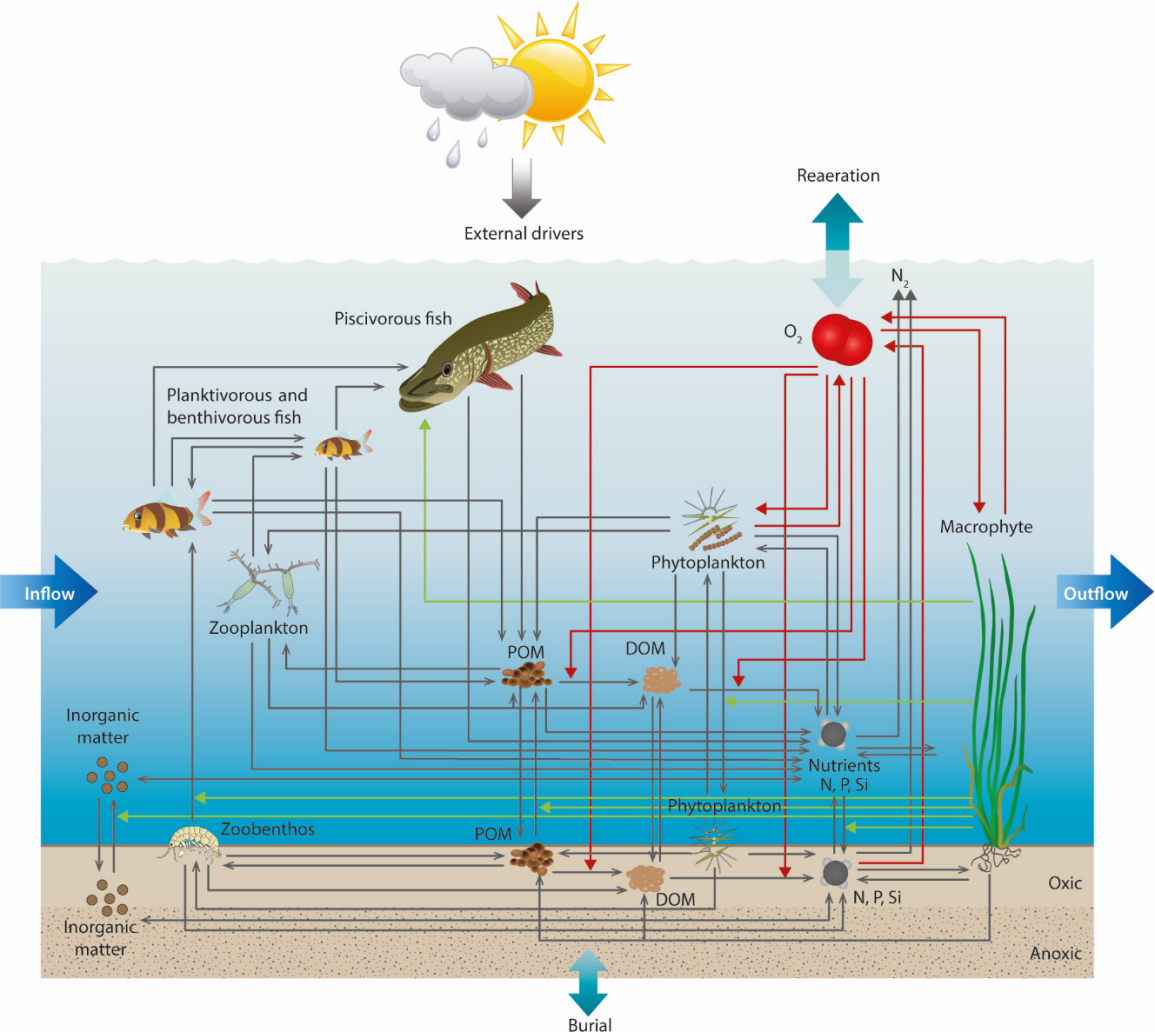
Conceptual figure of the aquatic ecosystem model FABM‐PCLake adapted from (Hu et al. 2016). Key state variables of FABM‐PCLake are shown and their interactions represented by gray arrows. Dissolved oxygen dynamics represented by red arrows and processes mediated by macrophytes are shown with green arrows.

FABM‐PCLake is a dynamic lake ecosystem model describing nutrient dynamics and interactions between multiple trophic levels within the water column and the top sediment (Fig. 3; Hu et al. 2016). The latest source code of FABM‐PCLake was downloaded in May 2019 with the modification of optional percentage winter fish kill (data *available online*).^6^
http://www.gitlab.com/FABM‐PCLake/pclake

^,^ ^7^
www.wet.au.dk/cases
 The model is a redesign of the widely applied aquatic ecosystem model PCLake (originally by Janse and Vanliere (1995)) within the Framework of Aquatic Biogeochemical Models (FABM), allowing the ecosystem model to be coupled with hydrodynamic models, in this case GOTM. This particular model coupling was chosen as it can represent the physical heterogeneity of lakes through a one‐dimensional hypsographic approach and explicitly describes higher trophic levels within a fully closed nutrient cycle.

Nutrient pools for nitrogen and phosphorus comprise ammonium (NH_4_), nitrate (NO_3_) and phosphate (PO_4_), dissolved organic matter (N‐ and P‐DOM), and particulate organic matter (N‐ and P‐POM) in the water column and sediment. Nitrogen and phosphorus are included in a sediment organic matter pool containing both POM and refractory organic matter (hereafter referred to as humus) and within all compartments of the food web. Important physicochemical processes are settling, sedimentation, resuspension, diffusion, adsorption, mineralization, and burial. Nitrogen and phosphorus cycles are fully closed. Primary production takes place in macrophytes and phytoplankton. Phytoplankton comprises three user‐defined groups (in this study diatoms, cyanobacteria, and other edible algae) differing in traits, some of the most important descriptors being growth rate, nutrient uptake rates, temperature and light optimum, physiological composition, and settling rates. Phytoplankton nutrient limitation is modeled by the Droop equation (Droop 1974) and growth rate depends on a tempo‐variable nutrient content of the phytoplankton group as well as light and temperature conditions. Chl *a* concentration is derived from a variable chl *a* to dry mass (DM) ratio constrained by the minimum and maximum ratios of each phytoplankton group and is higher in case of light limitation (Riegman 1985). Summation of all three phytoplankton chl *a* concentrations produces the total chl *a* concentration. The model also accounts for oxygen dynamics and includes processes such as reaeration, photosynthetically produced oxygen, and oxygen consumption by mineralization in the water column and sediment.

Higher trophic levels include zooplankton, detritivorous zoobenthos, juvenile and adult plankti‐benthivorous fish, and piscivorous fish. Zooplankton grazing depends on food availability and a filtering rate, which decreases hyperbolically with food availability and is modified by temperature. Zooplankton food preferences and edibility of food particles are user adjustable and in this study ranked as (with the highest preference first): diatoms, other edible algae, cyanobacteria, and POM. Juvenile plankti‐benthivorous fish predate on zooplankton, while adult plankti‐benthivorous fish feed on macrozoobenthos. Piscivorous fish predate on both age groups of plankti‐benthivorous fish and are to some extent dependent on macrophyte coverage.

Water temperature affects most processes in the model, both abiotic and biotic, by temperature‐dependent multipliers. Processes affected by temperature are reaeration at the atmosphere–water interface, diffusion across the water–sediment interface, as well as nitrification, denitrification, and POM and DOM mineralization modeled separately in the water column and top sediment. These processes are modified by exponential curves with a default temperature of 20°C. Seven biotic temperature multipliers amend the growth rates of phytoplankton, zooplankton, zoobenthos, plankti‐benthivorous, and piscivorous fish by Gaussian curves around an optimal temperature (with a customizable temperature deviation). The effects of temperature on macrophyte growth were modeled by two exponential functions representing effects of temperature on production and maintenance respiration. With a higher base value for maintenance respiration than production, the two exponential functions mimic an optimum temperature function controlling the growth of macrophytes (Janse 2005).

### Model input and forcing

Meteorological data for atmospheric forcing of GOTM were obtained from the European ECMWF Interim data set (Dee et al. 2011), which provides data at a three hourly resolution on air temperature (°C), air pressure (hPa), dew‐point temperature (°C), cloud cover (%), and wind speed components (m/s) in the north‐south and west‐east direction. Monthly averages of water inflow (m^3^/s) and nutrient concentrations (mg/L) were used as forcing data (see *Study site* for more information). Initial sediment values of inorganic matter, particulate organic matter, humus, absorbed phosphate, and the iron percentage of inorganic matter were estimated from sediment samples collected in 1992 and an estimated bulk density by Flindt et al. (2015). These initial values and parameters were also subject to changes duringthe calibration process and the sediment layer had a height of 10 cm.

### Model calibration and validation

GOTM‐FABM‐PCLake was calibrated and validated using two consecutive 7‐yr periods (1993–2007) against data following a 3‐yr warm‐up period (1990–1993). Calibration was based on comparison with observed water temperature and in‐lake water quality variables (Table 1). Phytoplankton was aggregated into the three user‐defined model phytoplankton groups (diatoms, cyanobacteria, and other algae) based on their taxa. This is the most comprehensive data set for calibration of a model in the PCLake family to date and the first time that zooplankton biomass is actively used in the calibration process.

**Table 1:** Procedure for calibrating Lake Hinge with seven steps focusing on different model processes and state variables.

Step	Focus on	Comparing model result against
1	physical processes	temperature
2	mineralization	dissolved oxygen (DO)
3	denitrification and nitrification	nitrogen (NO_3_, NH_4_, and TN)
4	P interactions in water column and sediment	phosphorus (PO_4_ and TP)
5	phyto‐ and zooplankton seasonal trends	chl *a*, phytoplankton composition and zooplankton dry mass
6	macrophyte dynamics	macrophyte coverage
7	all nutrient and plankton dynamics	steps 2–5

Note

The latter steps include calibrated parameters from the previous steps with narrowed ranges.

For model calibration, the auto‐calibration program ACPy was applied to perform an automatic global optimization of a selected subset of model parameters (program *available online*).^8^
www.bolding‐bruggeman.com/portfolio/acpy/
 ACPy applies the parallel direct search method Differential Evolution (Storn and Price 1997) to estimate the most optimal choice of model parameter values within predefined, parameter‐specific ranges based on optimization of a Maximum Likelihood multi‐objective function. This allows a larger search for optimal parameter values in the global parameter space than previously achieved by manual trial‐and‐error calibration. For an example of program usage on a hydrodynamic lake model with five parameters calibrated, see Moras et al. (2019).

The calibration procedure was based on a bottom‐up approach including seven consecutive steps with individual selected parameters and state variable(s) for each step (see Table 1). The parameter selection was inspired by sensitivity analysis by Janse et al. (2010) and Nielsen et al. (2014) and experience in FABM‐PCLake model usage. During each calibration step (Table 1), thousands of model runs were executed via ACPy, ultimately producing an optimal value with a narrower range of the selected parameters to increase model performance. The narrowed parameter ranges were then applied in the next step of the calibration procedure. Model performance for the daily output of each state variable was evaluated by calculating the coefficient of determination (*R* ^2^), relative mean absolute error (MARE), relative error (RE), root mean square error (RMSE), and bias (BIAS) to describe both the correlation between observed and modeled values, while also taking into account the bias or offset in the modeled values as recommended by Bennett et al. (2013). To include the entire dynamics of increase in macrophyte coverage, macrophyte‐specific parameters were calibrated based on both the calibration and validation periods against depth‐distributed macrophyte coverage (step 6, Table 1). Then, calibration on all plankton and nutrient dynamics previously calibrated (step 7) continued until ranges of selected parameters (see Appendix S1: Table S1) were markedly decreased and model error could no longer be appreciably reduced (Table 2). The initial list of parameters to be calibrated included 60–70 parameters known to affect the water quality state variables in the model. As the auto‐calibration tool allowed for a faster calibration process than previous manual calibrations, other parameters that had not previously been included in a calibration process were tested to check if the parameters markedly decreased model error; if so, the parameters were added to the list of calibrated parameters, resulting in a total of 143 parameters calibrated for the lake model for Lake Hinge.

**Table 2:** Coefficient of determination (*R* ^2^), mean absolute relative error (MARE), relative error (RE), root mean square error (RMSE), and percentage bias (BIAS) between modeled output and observations for daily time steps separated in calibration (1993–2000) and validation (2000–2007) periods.

Variable	Calibration (1993–2000) | Validation (2000–2007)				
	*R* ^2^	MARE	RE	RMSE	BIAS
Temperature (°C)	0.96|0.98	0.21|0.15	0.13|0.11	1.99|1.68	−2.0|2.3
DO (mg O_2_/L)	0.18|0.21	0.17|0.11	0.14|0.10	2.24|1.49	6.0|5.1
Chl *a (*µg/L)	0.43|0.43	0.66|0.68	0.41|0.44	48.1|46.7	−6.6|6.0
TP (mg P/L)	0.24|0.32	0.32|0.31	0.28|0.26	0.05|0.04	−5.1|8.0
TN (mg N/L)	0.89|0.83	0.30|0.25	0.21|0.20	0.81|0.60	10.8|9.70
PO_4_ (mg P/L)	0.14|0.32	0.91|1.19	0.62|0.63	0.01|0.01	1.1|7.8
NO_3_ (mg N/L)	0.90|0.89	3.96|8.70	0.30|0.33	0.76|0.56	16.7|19.1
NH_4_ (mg N/L)	0.35|0.20	5.73|1.57	1.26|0.71	0.07|0.07	95.9|15.5
Zooplankton (mg DM/L)	0.36|0.14	1.42|1.22	0.71|0.70	0.72|0.96	−16.9|−30.1
POM (mg DM/L)	0.38|0.42	0.72|0.43	0.40|0.36	5.82|5.21	2.6|1.0
Macrophytes (% coverage)	0.09|‐‐‐‐‐‐	–|–	1.34|‐‐‐‐‐‐	4.95|‐‐‐‐‐‐	17.5|‐‐‐‐‐‐

Notes

Calibration data set for particulate organic matter (POM) contained 3 yr of data. Macrophytes were calibrated on the entire model simulation period and therefore no validation statistics could be calculated (represented by dashes). MARE was not calculated for macrophytes. DM, dry mass.

### Bifurcation analysis with differing external phosphorus load scenarios

**Fig. 4:**
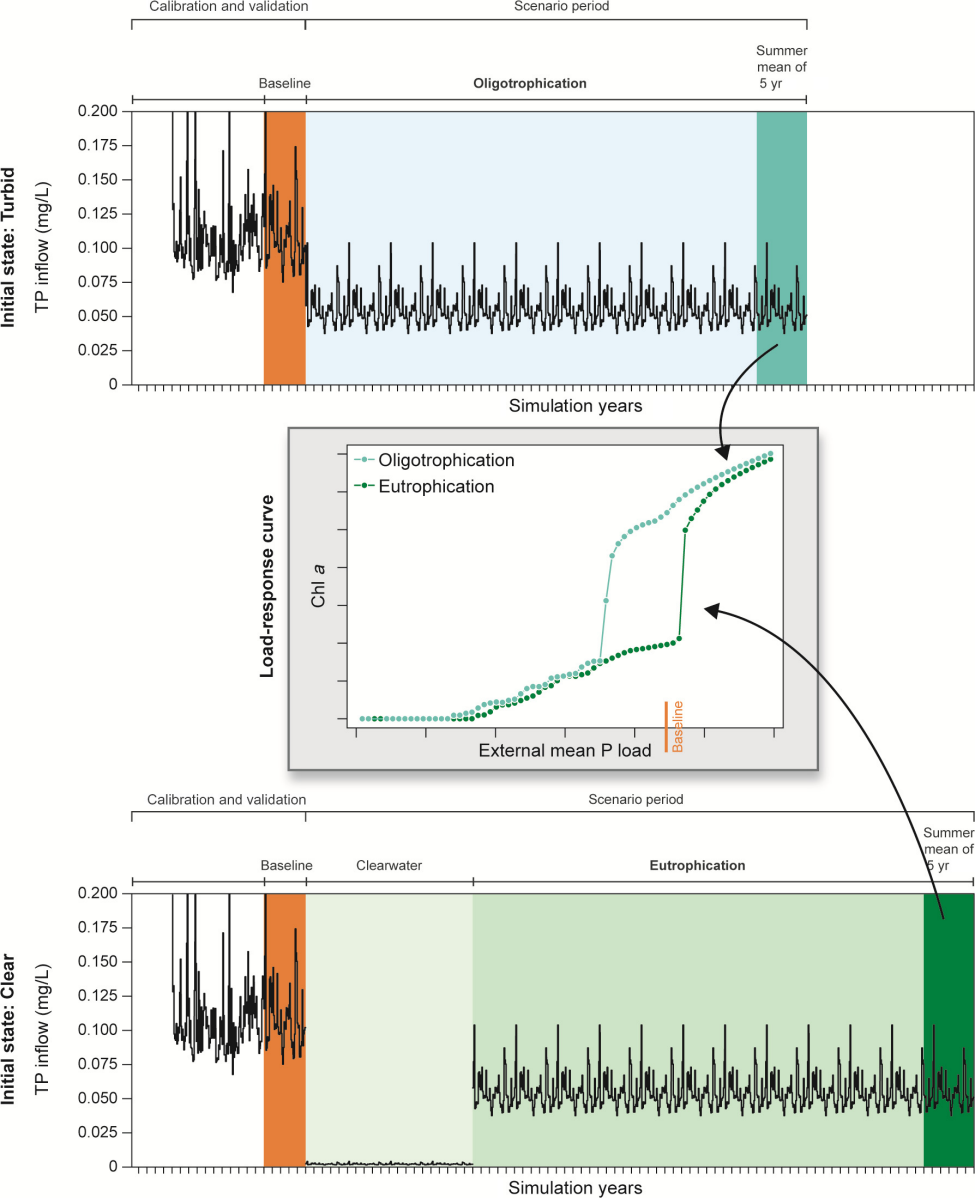
Conceptual depiction of the bifurcation analysis with 75 different external P load scenarios at two different initial ecosystem states, turbid and clear‐water (top and bottom plot, respectively) and their corresponding load‐response curve (gray center plot). A five‐year baseline from the validation period (2001–2006, orange band) was used to calculate new external phosphorus loadings in increments of 2% within a −98% to +50% range. Oligotrophication scenarios (top plot) were simulated with 12 repetitions of baseline (60 yr in total, light blue band) for all new external loadings, and summer means of the last five years of the simulation period (dark blue band) were extracted. Eutrophication scenarios (bottom plot) were simulated with a 98% P load reduction for 20 yr to establish a “clear‐water state” in the lake (light green band) following 12 repetitions of baseline (60 yr in total) (light green band) with all new external loading increases, and summer means of the last five years of the simulation period (dark green band) were extracted. Extracted summer means from both scenarios were then ranked and plotted according to their external mean P load in a load‐response curve (gray center plot). Baseline load in load‐response curve represents the 5‐yr external P load mean.

To assess the effects of external phosphorus loads on lake ecosystem state, we applied a bifurcation analysis to the validated lake model for Lake Hinge (Fig. 4). In this, each scenario simulation was initialized from both a turbid water state and a clear‐water state to also assess the possibility of any hysteric effects in the model for Lake Hinge, i.e., any effects of initial ecosystem state on the scenario outcomes. The five‐year period 2001–2005 was chosen as a baseline as this would provide some degree of accounting for year‐to‐year variability in the simulated scenarios (orange range in Fig. 4). Scenarios for external phosphorus load changes were implemented in increments of 2% within a −98% to +50% range based on baseline external loading and forcing conditions (totaling 75 different external load scenarios). The 5‐yr scenario period was then looped 12 times, creating a 60‐yr scenario simulation period with a new external P load.

The ecosystem state of Lake Hinge at baseline was turbid, so to simulate oligotrophication a time series of forcing conditions for each external load reduction scenario (−98% to 0% of baseline P load) was created by adding the 60‐yr external load scenario after the baseline period. To also assess the effects of a P load increase on an initial turbid ecosystem state, a time series of forcing conditions of P load increase scenarios (+2% to + 50% of baseline P load) was created. This resulted in 75 different external load scenarios with corresponding time series of forcing conditions that spanned warm‐up, calibration, and validation periods and a 60‐yr scenario of new external load change (1990–2065). Sixty years has been shown to be an adequate duration to achieve a state close to stable within the lake for each external load scenario. For an example of an oligotrophication scenario for TP inlet concentration, see top plot in Fig. 4. To simulate eutrophication from an initial clear‐water state, the first 20 yr of the −98% P load reduction scenario were added after the baseline period to achieve a clear‐water state in the simulation (light green range in bottom plot in Fig. 4), followed by adding of the 60 yr of external P load scenarios (−98% to +50% from baseline P load; green range in Fig. 4). For an example of an eutrophication scenario for TP inlet concentration, see bottom plot in Fig. 4.

Mean summer (May to September, both months included) values of water quality variables, mean benthic area‐weighted macrophyte coverage and density at higher trophic levels, and the maximum depth limit of macrophytes in August for the last 5 yr of all the 150 P load change scenarios for oligo‐ and eutrophication were extracted, ranked, and plotted according to their external mean P load to create load–response curves (center plot in Fig. 4). In other words, each point in the load‐response curve represents a 5‐yr summer mean of a specific state variable extracted from one out of 75 external P load change scenario simulations with either a turbid or a clear‐water initial state. To determine the macrophyte depth limit, a threshold for macrophyte coverage was set at 0.1%, which was the amount of biomass in the model that roughly corresponded with the observed depth limit in the best calibration.

## Results

### Calibration and validation of model for Lake Hinge

**Fig. 5:**
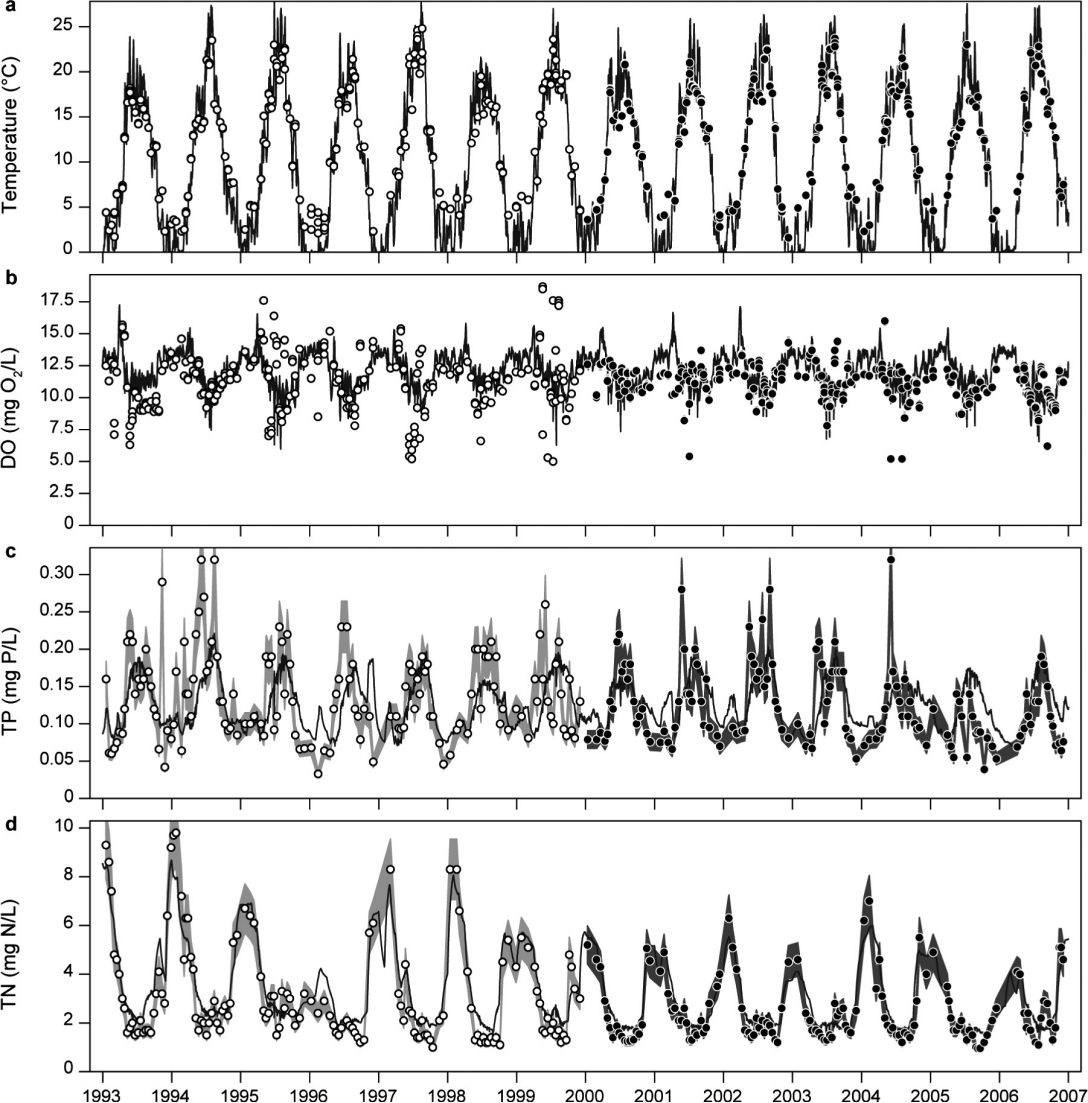
Simulated model values (black lines) against observed values in the calibration and validation period (white circles and light gray band, black circles and dark gray band, respectively) for (a) temperature, (b) dissolved oxygen (DO), (c) total nitrogen (TN), and (d) total phosphorus (TP). TN and TP observations include 15% error band to indicate uncertainty related to observations.

Simulated water temperatures spanned the observed water temperature range of approximately 0°–25°C and showed excellent agreement with observed data during both the calibration and the validation period by capturing the timing and inter‐annual variations (Table 2; Fig. 5a). Simulated DO generally increased in the winter periods, being around 12–17 mg O_2_/L, which was in correspondence with observations. During the summer periods, the model generally overestimated DO, although the simulations spanned the recorded summer range of around 5–10 mg O_2_/L. The model captured seasonal dynamics of DO reasonably well with a low relative error (RE ≤ 0.13) but was in some years not able to explain the magnitude of summer minima or the high variability in the observed data (Table 2; Fig. 5b). This resulted in overall low *R* ^2^ values of 0.18 and 0.21 for the calibration and validation period, respectively.

**Fig. 6:**
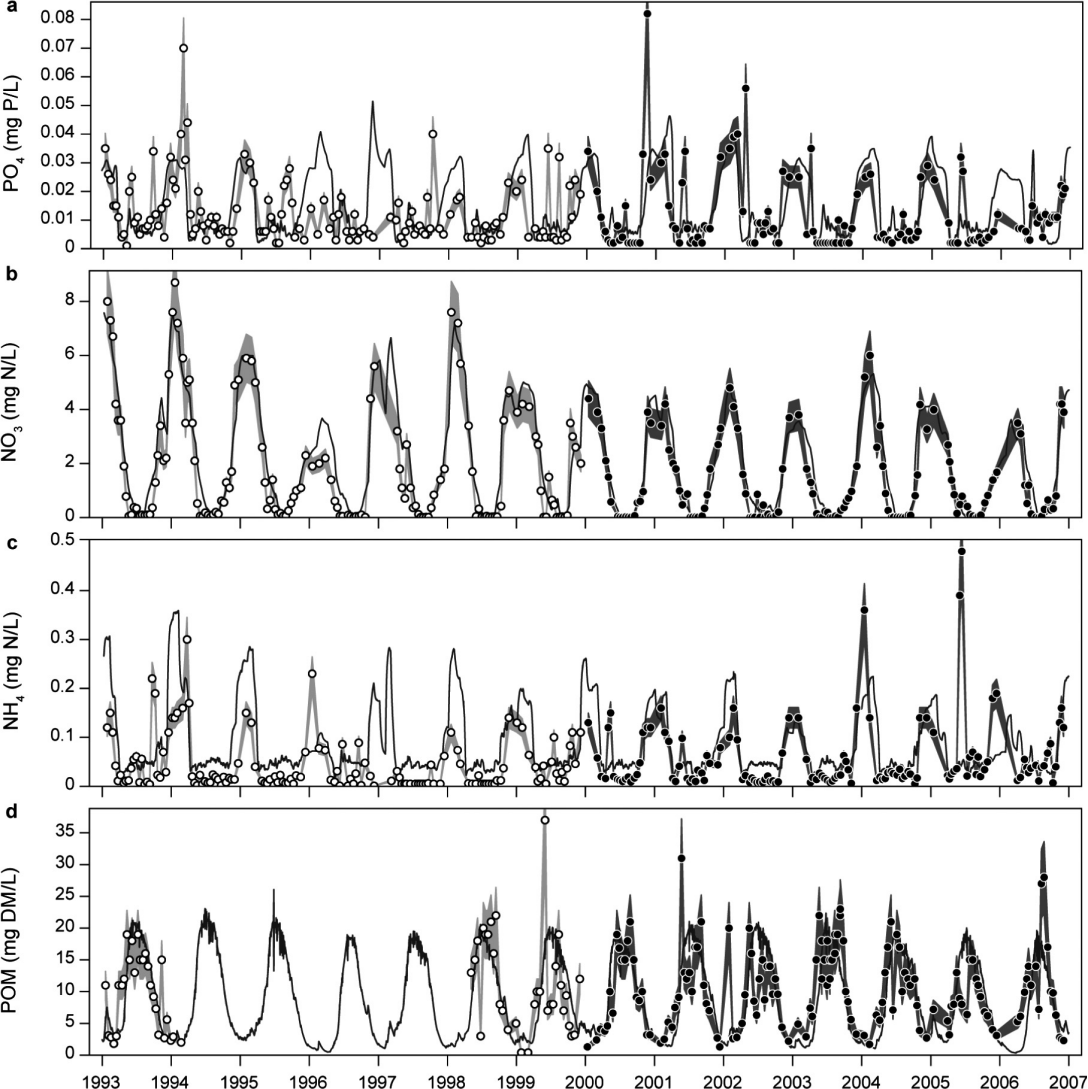
Simulated model values (black lines) against observed values in the calibration and validation period (white circles and light gray band, black circles and dark gray band, respectively) for (a) phosphate (PO_4_), (b) nitrate (NO_3_), (c) ammonium (NH_4_), and (d) particulate organic matter (POM). All observations include a 15% error band to indicate uncertainty related to observations. DM, dry mass.

Simulated TN and NO_3_ concentrations excellently described the timing and magnitude of observed values and the inter‐annual variation in both the calibration and the validation period with all *R* ^2^ values being above 0.8 and BIAS below 20% (Table 2; Figs. 5d, 6b). Simulated NH_4_ concentrations generally showed a fair agreement with observations (*R* ^2^ = 0.35 and 0.2), though in some years the model did not capture the magnitude of winter concentrations (Table 2; Fig. 6c). The simulated dynamics of TN were largely driven by in‐lake nitrate in winter and spring, which contributed at least 60% of the TN, with in‐lake nitrate peaks of 4–8 mg N/L. Simulated NH_4_ concentrations also peaked in winter to spring but always contributed only a small fraction of TN, with peaks in the range of 0.1–0.4 mg N/L. In summer, the model overestimated summer concentrations of NH_4_ in the calibration period but simulated the occurrence of NO_3_ depletion as indicated by observations. Simulated summer TN was mainly made up of POM‐N and N stored in the phytoplankton pool (20–25% and 15–20%, respectively), DOM‐N being the major fraction in late summer and autumn (40–50%) and nitrate the main fraction in winter and spring (60–90%).

The model succeeded in capturing the overall seasonal patterns and magnitudes of TP in most years despite the high variation in observations; all *R* ^2^ values were between 0.24–0.32 and BIAS range between −5.1 and 8% (Table 2; Fig. 5c). In most years of the calibration period, the model did not capture the summer peaks of TP even though seasonal timing and magnitude were described well by the model. Seasonal timing and magnitude of PO_4_ were captured well by the model (*R* ^2^ between 0.14 and 0.32); a few sampled phosphate peaks were not captured, however (Table 2, Fig. 6a). Simulated winter and spring TP was mainly made up of the phosphorus fraction of POM (P‐POM) and PO_4_ (40–60% and 20–30%, respectively), summer TP was composed of DOM‐P (40–60%) as well as POM‐P and phytoplankton‐P (20–30% and 10–25%, respectively), and autumn TP was mainly composed of DOM‐P (50–60%).

**Fig. 7:**
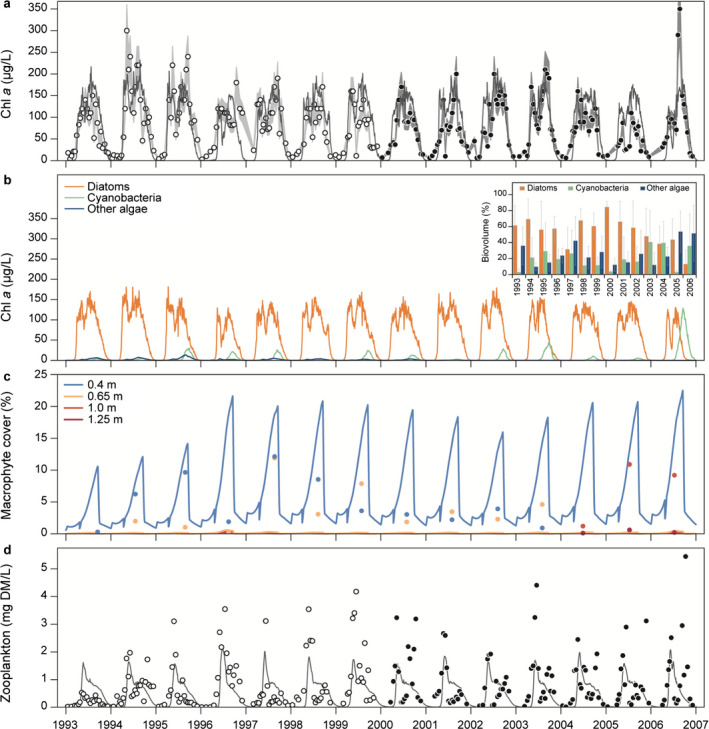
Simulated model values for (a) chl *a*, (b) diatom, cyanobacteria, and other algae chl *a*, (c) macrophyte coverage, and (d) zooplankton biomass. (a) Simulated total chl *a* concentrations against observed values in calibration and validation period (light gray band and dark gray band, respectively). All observations include a 20% error band to indicate uncertainty related to observations. (b) Simulated phytoplankton chl *a* concentrations for each phytoplankton group (diatoms [orange], cyanobacteria [blue], and other algae [light blue]) with observed percentage of summer mean biovolume (mean and standard deviation) in inset. (c) Simulated macrophyte percent cover for depths 0.4 m (blue), 0.65 m (orange), 1.0 m (red), and 1.25 m (dark red) against observations (circles in corresponding color). (d) Simulated zooplankton biomass against observed values in the calibration and validation period (white circles and black circles, respectively).

The simulated dynamics of chl *a* concentrations captured the seasonal timing and magnitude of observed data well in both the calibration and the validation period with all *R* ^2^ values being above 0.4 and BIAS below ±7% (Table 2, Fig. 7a). The model simulated a clearer‐water period every year, which corresponded well with both the observed and simulated increases in zooplankton biomass (Fig. 7d), although the simulated timing of the spring bloom and the clearer‐water period occurred one to three weeks earlier than indicated by the observations in about half of the simulation years. Every spring bloom was composed of the simulated diatom group, and in most years when filamentous cyanobacteria occurred (mainly represented by *Anabaena* sp.), the model was able to simulate a late summer to fall cyanobacteria peak. However, observations show that most late summer peaks were dominated by diatoms (mainly *Aulacoseira* sp.), which the model did capture (Fig. 7b).

The simulations of zooplankton corresponded fairly well with the timing of the late spring biomass peak (*R* ^2^ between 0.14 and 0.36), but the peak concentrations were generally underestimated (BIAS between −17% and −30%) (Table 2, Fig. 7d). During summer and fall in most simulation years, the model simulated a continuous decrease of zooplankton biomass concentrations due to fish predation and reduced food availability, whereas observations show a zooplankton biomass peak in fall in some years, especially in the validation period. The amount of POM in the water column was found to affect plankton dynamics as zooplankton grazed on POM, and during high resuspension events POM affected light conditions for phytoplankton. Therefore, correspondence between model simulations and observations was strived for during the calibration process. This was accomplished as the model described well both the timing and magnitude of POM concentrations with *R* ^2^ values approximately 0.4 and low percentage bias (Table 2, Fig. 6d). In both simulation periods, zooplanktivorous and benthivorous fish represented approximately 65% and 35% of the August mean of the total fish biomass, respectively, piscivorous fish constituting only 1–2% (data not shown). Three fish surveys in the simulation period in late summer all estimated approximately 90% zooplanktivorous and benthivorous fish and 10% piscivorous fish of the total fish biomass (Johansson et al. 2016).

The model was able to approximately describe the level of macrophyte coverage at 0.4 m in late summer in 1994–1998, though overestimated the coverage in the rest of the simulation years (Fig. 7c). However, for coverage deeper than 0.65 m the model did simulate low coverage corresponding to observations in most years. Generally, the model only adequately captured the observed inter‐annual variation in the validation period (*R* ^2^ = 0.09; Table 2, Fig. 7c). The model was not able to simulate the increase in macrophyte coverage observed at 1 m in the last 2 yr of the validation period; however, very low coverage was simulated for depths deeper than 1 m, which corresponded with observations for the entire simulation period. A general depth limit at 1.2 m across the simulation period corresponded to a modeled coverage of 0.1 % with no considerable growth below the depth limit due to limited light conditions.

### Bifurcation analysis with P load reduction scenarios

**Fig. 8:**
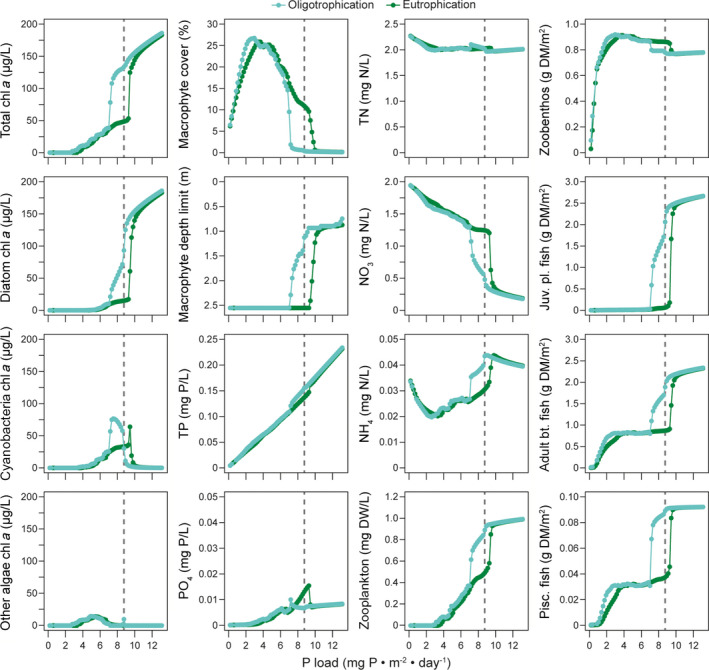
Load‐response curves from bifurcation analysis of summer means of the last 5 yr in an oligotrophication (green circles) and a eutrophication (blue circles) scenario for a −98% to + 50% change in external P load for various state variables. Dashed vertical line represents the calibrated state (no external P load change). Juv., juvenile; pl., zooplanktivorous; bt, zoobenthivorous; Pisc., piscivorous.

A bifurcation analysis was undertaken with the calibrated and validated model for Lake Hinge to estimate the effects of external phosphorus load reductions on the ecosystem state of the lake. The oligotrophication scenario, where external P loads were changed from the baseline period (2001–2005), predicted an ecosystem state change from a turbid, phytoplankton‐dominated state to a clearer‐water, macrophyte‐dominated state at 16–20% reductions of the external P load (corresponding to an external load of 7–7.4 mg P·m^−2^·d^−1^) (Fig. 8). Low P load reductions generally favored cyanobacteria in fall because summer mean cyanobacteria biomass increased (cyanobacteria DW plot in Fig. 8) and the phytoplankton community composition thereby shifted. Higher trophic levels such as zooplankton and fish reacted to the reduced food availability and decreased as well.

With a further decrease in P load, maximum macrophyte coverage gradually increased to ~26% at 3.0 mg P·m^−2^·d^−1^ (74% P load reduction) accompanied by a gradual summer mean chl *a* decrease. Only zoobenthos biomass did not clearly react to decreased P load as food availability was determined by organic matter in the sediment, and a rather constant zoobenthos biomass maintained biomass levels of benthivorous and piscivorous fish until a decrease occurred at P loads below 1 mg P·m^−2^·d^−1^. TP and PO_4_ summer mean concentrations generally decreased with P load reductions, whereas phosphate peaked within the hysteresis range. In general, TN summer mean concentrations were stable until an increase appeared at P loads below 3–4 mg P·m^−2^·d^−1^, while NO_3_ summer mean concentrations increased with decreasing P loads.

During the 20‐yr clear‐water period in the eutrophication scenario, macrophytes established and grew at all lake depths. With increasing P loads (0–3.5 mg P·m^−2^·d^−1^ load range), submerged biomass and coverage gradually peaked and reached the level of the oligotrophication scenario and then gradually decreased to approximately 12% coverage in the P load scenario with no further reduction. In the eutrophication scenario, the macrophyte depth limit did not respond to changes in P loads before the external P load increased to a level above the baseline P load.

**Fig. 9:**
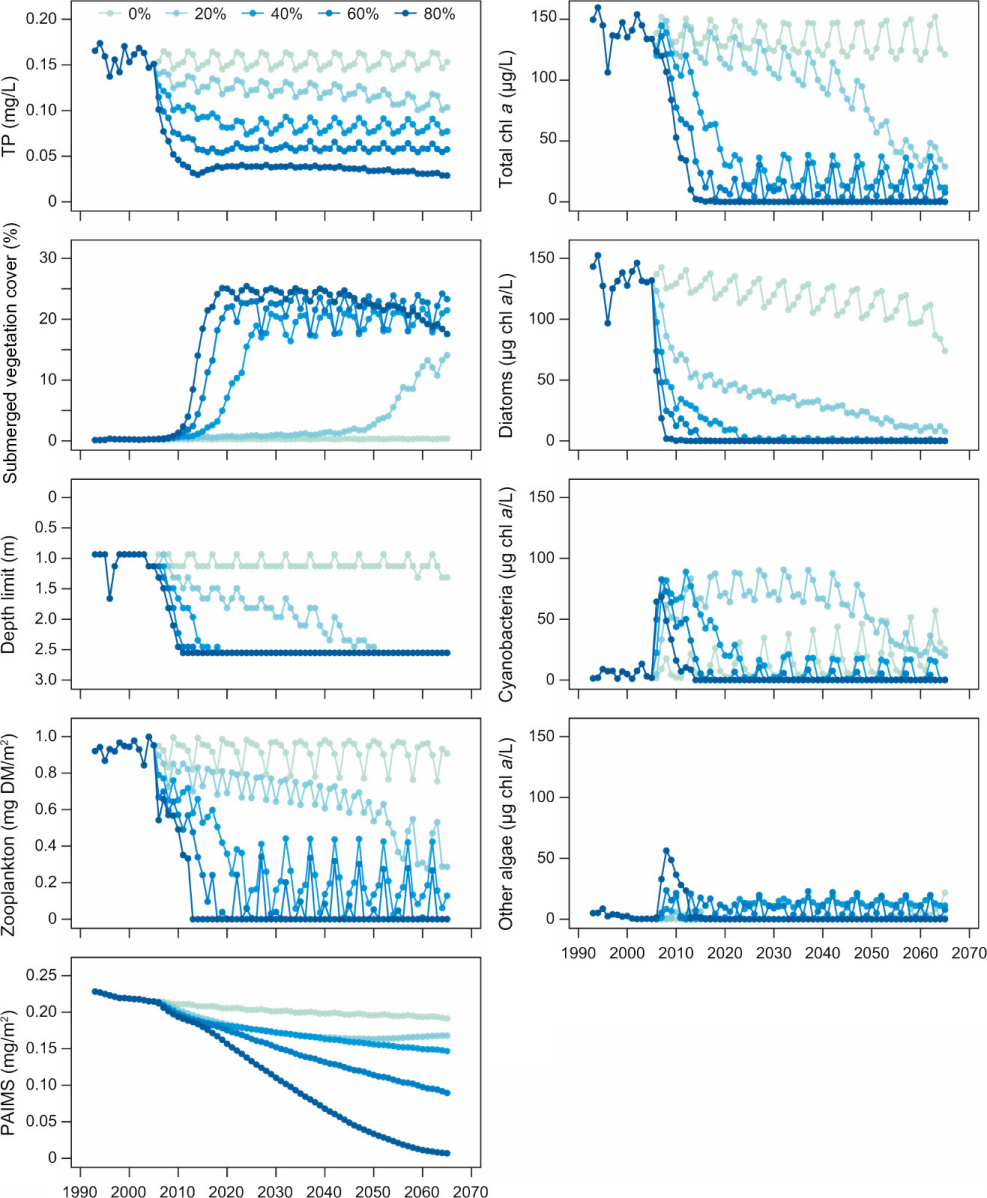
Time series of summer means from five oligotrophication scenarios with an 80% to 0% P load reduction during the calibration (1993–2000), validation (2000–2007), and scenario (2005–2065, a looped 5‐yr period) periods for TP, macrophyte coverage and depth limit, chl *a* concentrations for total phytoplankton and individual phytoplankton groups, zooplankton biomass, and phosphorus bound to inorganic matter (PAIMS).

The bifurcation analysis showed a clear difference in the ecosystem response of most state variables across all trophic levels between the oligotrophication and the eutrophication scenarios (Fig. 8). The hysteresis effect was within the P loading range of 7–10 mg P·m^−2^·d^−1^. Not all scenario simulations reached a steady state for all state variables. An example is phosphate adsorbed to inorganic matter in the sediment (PAIMS), which for most P load reductions did not fully stabilize (Fig. 9, PAIMS). Another example is the gradual increase of cyanobacteria peaks in fall in the no further reduction scenario; thus, after 60 yr of simulation cyanobacteria had tripled their biomass compared with the validation period.

The bifurcation analysis showed a correlation between the percentage reduction and the lag time for an ecosystem state change (Fig. 9). In the oligotrophication, 20% reduction scenario, no apparent change in macrophyte coverage occurred in the first 40 yr of simulation even though the chl *a* level steadily decreased and the macrophyte depth limit increased. As the depth limit reached maximum lake depths, the macrophyte coverage started to increase with a clear drop in chl‐*a* concentrations as a result.

## Discussion

### Effect of depth heterogeneity on simulated macrophyte dynamics and ecosystem state change

A gradual lake ecosystem response was observed in the P loading reduction scenarios for Lake Hinge and this did not indicate resilience as each reduction in the P load induced an ecosystem response for most state variables (e.g., decrease in chl *a*, Fig. 5) as also recorded on contemporary data from several other Danish shallow lakes (Jeppesen et al. 2005, 2007). Even though the bifurcation analysis demonstrated a hysteretic effect, the transition between the turbid to the clear‐water state was less sudden than in bifurcation plots previously obtained with PCLake (e.g., Janse et al. 2008).

To evaluate the effect of FABM‐PCLake including a water column and a sediment layer in each water layer, we compared our model results for Lake Hinge with the 0D PCLake presented in Janse et al. (2008; see Fig. 2 for a comparison between the 0D and 1D depth and sediment configuration). The 0D PCLake model was calibrated against nutrients, transparency, chl *a*, and vegetation data on 43 European lakes (mainly Dutch; Janse 2005) and has not previously been calibrated for Lake Hinge. The default parameterization of the 0D PCLake model was not able to capture increases in macrophyte coverage below 20% (Janse 2005). As the critical P load was originally defined by at least 20% macrophyte coverage, PCLake showed an “all or nothing” response to regime shifts (Janse 2005, Mooij et al. 2010). GOTM‐FABM‐PCLake simulations for Lake Hinge with all default 0D PCLake parameter values resulted in high macrophyte coverage in the shallowest areas in the first three simulation years, and after four additional years the deepest parts of Lake Hinge were also colonized above 20% in late summer (data not shown). The depths of the later years’ colonization correspond approximately to the depths deeper than the Lake Hinge 0D PCLake sediment depth of 1.2 m (Fig. 2). This supports the hypothesis that by simulating light distribution through the water column and its associated benthic area in each water layer, the modeled submerged macrophytes initially (re)established more easily in the shallower areas, promoting further colonization in the deeper parts of the lake and not an all or nothing effect.

This study therefore corroborates the hypothesis that by including depth heterogeneity in the lake model, ecosystem state responds more gradually to external P load changes due to, among other factors, allowing faster reestablishment of macrophytes in the littoral zone. Since this model study is case‐specific, the model’s prediction of a more gradual ecosystem state response may be so too. However, more general models investigating heterogeneity in relation to regime shifts have also concluded that when including spatial heterogeneity, the overall ecosystem response is more gradual (Nes and Scheffer 2005).

Specific 0D PCLake model predictions for Lake Hinge were based on input of lake characteristics such as area, depth, fetch, marsh part, sediment type, inflow, and retention time in the general 0D PCLake and a subsequent bifurcation analysis. A threshold P load for restoration of Lake Hinge was predicted to be 8.1 ± 4.1 mg P·m^−2^·d^−1^ at which Lake Hinge would show a stable clear‐water state with at least 20% macrophyte coverage after 20 yr (Janse 2005, Janse et al. 2008). The bifurcation analysis with 1D GOTM‐FABM‐PCLake predicted a 20% macrophyte coverage at the external P load of approx. 6 mg P·m^−2^·d^−1^, which was within the predicted clear‐water state P load range of the 0D PCLake. Overall, the 1D GOTM‐FABM‐PCLake thereby supported the magnitude of P load threshold range required to achieve a clear‐water state, as indicated by the 0D PCLake, although 1D GOTM‐FABM‐PCLake was able to predict a gradual ecosystem state change.

### Are clear‐water feedback mechanisms too dominant in FABM‐PCLake?

Macrophytes promote clear‐water conditions by several mechanisms that contribute to a positive feedback loop between macrophytes and water clarity (Jeppesen et al. 1998, Søndergaard et al. 2016). Several of these mechanisms are built into the model structure of PCLake and FABM‐PCLake: macrophytes suppress phytoplankton growth via competition for light and nutrients, reduce resuspension of particulates, and promote piscivorous fish growth by a dependency function (Janse 2005, Hu et al. 2016). Also, the assimilation of piscivorous fish is linked to the macrophytes and therefore helps to promote a faster shift between turbid and clear‐water states by enhancing the predation on coarse fish when macrophytes appear. Although piscivorous fish dependency on macrophytes has been greatly reduced in the calibrated model (parameters cCovVegMin and hDVegPisc, see Appendix S1: Table S1), the bifurcation analysis clearly demonstrated a hysteresis effect (Fig. 8).

There has been some debate over and criticism of the concept of regime shifts, with contemporary data suggesting that some systems often show a gradual rather than an immediate temporal transition when a lake shifts from a turbid to a clear state and vice versa (Sayer et al. 2010*a*, Capon et al. 2015). It is therefore also reasonable to question the conceptual models of PCLake and FABM‐PCLake, both of which have been developed to enable simulations of regime shifts. It is clear, however, that there are strong ecosystem feedbacks of macrophytes and phytoplankton, which is well documented (Jeppesen et al. 1998, Bolpagni et al. 2014, Søndergaard et al. 2016), and these feedbacks should therefore also be included in a shallow lake ecosystem model. A sensitivity analysis of the level of the critical nutrient load by 0D PCLake indicates that the macrophyte parameters are particularly sensitive (Janse et al. 2008). This is also supported by our results, the experience gained from calibrating the model, and a bifurcation analysis conducted in a different study using a modified, recalibrated version of PCLake, including a temperature optimum for macrophytes and an empirical description of periphyton shading (Hilt et al. 2018) (hereafter referred to as PCLake with periphyton shading). The adjusted PCLake with periphyton shading had critical P loads for oligotrophication and eutrophication of 1.06 mg P·m^−2^·d^−1^ and 1.3 mg P·m^−2^·d^−1^, respectively, with a decrease in chl‐*a* of approx. 15 µg/L (Hilt et al. 2018) compared with the critical P loads of the original PCLake model of 0.9 and 3.0 mg P·m^−2^·d^−1^, respectively, with a decrease in chl *a* of 40–70 µg/L (Janse et al. 2008). Thus, the hysteresis range (the load range between oligo‐ and eutrophication) was limited in both P load range and ecological response (i.e., chl *a* concentration) by including periphyton shading and a temperature optimum for macrophytes in the conceptual model. In this way, a structural change in the conceptualization of light competition and the description of macrophytes (the original PCLake vs PCLake with periphyton shading) had a marked influence on the P load threshold and level of hysteresis. This points to the facts that external P load scenarios are sensitive to parameterization of macrophytes and that the conceptual description of macrophytes is important for the overall ecosystem state response.

Experiences from a series of lakes worldwide indicate that it generally takes 10–15 yr for the P pool in the sediment to stabilize after reduced loading (Jeppesen et al. 2005*b*); some lakes may, though, show a much longer response time (Søndergaard et al. 2001). The prolonged P sediment pool lag phase simulated by the FABM‐PCLake model affected other ecological responses as well, for example the gradual decrease in the summer sediment phosphate release and the gradual increase in cyanobacteria fall peaks (Fig. 9). The trajectory of the P pool was expected as the initial P pool was set to incorporate effects of a higher loading in the past. However, the absolute increase in cyanobacteria biomass was not expected as several studies have only shown an increase in the ratio of community composition and not an absolute biomass increase in cyanobacteria during oligotrophication events (Jeppesen et al. 2005*a*, *b*). The calibration process demonstrated a need for changing the P characteristics (e.g., P affinity and P maximum uptake rates) of the three phytoplankton groups in order to simulate changes in phytoplankton composition. The increase in cyanobacteria biomass points to sensitivity of the phytoplankton P characterization that should be taken into account in future bifurcation sensitivity analyses.

Following P load reductions, typically both TP and TN concentrations decrease in shallow lakes (Jeppesen et al. 2007). With improved light conditions at the sediment surface, benthic primary production increases nutrient uptake rates (Vadeboncoeur et al. 2003) and oxygen concentrations at the water‐sediment interface, while decreased mineralization of organic material (less settled) also improves sediment oxygen conditions (Jensen and Andersen 1992). The model did capture the shift from pelagic to benthic primary production in response to changed N dynamics at mid‐range P loads with an increase in the oxic fraction of the sediment and a decrease of the nitrogen fraction bound in phytoplankton (data not shown). This maintained stable TN levels since NO_3_ concentrations increased with P load reductions (Fig. 8). Nitrate would not be expected to increase even though there was a lower amount of NO_3_ taken up by phytoplankton due the now lower TP, but instead denitrified, thereby keeping the NO_3_ at the same level as before the P load reduction (Søndergaard et al. 2005*a*). This indicates that N dynamics in the model were not parameterized sufficiently well to account for increased NO_3_ loss with decreasing TP concentrations.

As estimating a P load threshold with the bifurcation analysis proved sensitive to both model structure and parameterization, we recommend applying bifurcation analysis in an ensemble context. This will help account for and visualize parameter uncertainty when multiple model instances, representing somewhat different model parameterizations, are applied in the bifurcation analysis. Use of multiple model instances also addresses the potential of non‐uniqueness (also known as equifinality) of the modeled system: several combinations of different parameter values may result in equally satisfactory model performances in the calibration process, but potential divergences may occur in the simulated outcome of future scenarios that are beyond the calibrated domain (Beven 2006). For a lake example of multiple model instances to account for equifinality, see (Nielsen et al. 2014).

### Model calibration and performance

Overall, the model reproduced well seasonal and inter‐annual variations in water temperature, nutrient concentrations, and chl *a*, but some discrepancies occurred between model outputs and observations for dissolved oxygen and higher trophic levels (Table 2). Compared with a meta‐analysis of 153 aquatic modeling studies (Arhonditsis et al. 2006), model performance (*R* ^2^ values) for temperature, chl‐*a*, and inorganic nutrients was within the reported performance range; however, many of these studies did not include as many state variables and as long time series as this study. For TN and TP, model performance (RMSE) was comparable with several model studies of lakes using DYRESM‐CAEDYM in New Zealand and Denmark (e.g., Burger et al. 2008, Trolle et al. 2008, Özkundakci et al. 2011).

The model reproduced a general level of macrophyte coverage for the entire simulation period, but was not able to reproduce specific water layer macrophyte dynamics (Fig. 7c). This should likely not be expected of the model as there are inherent uncertainties related to the observation of macrophyte coverage (Søndergaard et al. 2016); however, there is yet to be an investigation providing an estimate of these uncertainties. The modeled macrophyte dynamics should be interpreted with caution as the model did not succeed in capturing the increase in coverage and depth limit in the last two years of the simulation period, although as the macrophyte coverage increased with decreased TP external loading (Figs. 8, 9), the general behavior of the modeled macrophytes mimics re‐establishment of macrophytes after reduced P loading as observed in Danish lakes (Jeppesen et al. 2007).

The calibration process revealed sensitivity of DO, phytoplankton, and PO_4_ dynamics to physical parameters describing turbulence and wind‐induced mixing. Therefore, to make the simulation of particulate resuspension more reliable, observations of particulate matter were included in the calibration, and the calibrated model accomplished a good simulation of POM (Fig. 6). To further improve the description of particulate resuspension in FABM‐PCLake, a critical shear stress parameter for each phytoplankton group, POM, and inorganic matter, as in the DYRESM‐CAEDYM model (Hipsey et al. 2010), should preferentially be included.

The conceptual description of zooplankton biomass included one functional group with no zooplankton life history traits. This likely constrained the model, which was not able to reproduce the observed mid‐summer zooplankton peak dominated by the genus *Daphnia* in later simulation years. The need to include zooplankton life history traits to effectively describe zooplankton dynamics in a modeling context has been pointed out by several authors (Arhonditsis et al. 2006) and has also been implemented in some marine models (Fennel and Neumann 2001). Still, the model captured a mid‐summer clearer‐water period caused by zooplankton grazing, though for some years the timing of the grazing was simulated one to three weeks too late. The model reproduced zooplankton dynamics adequately for the purpose of this study, but the results should be interpreted with some caution. This also holds true for the higher trophic levels as the representation of the fish groups is probably too coarse (Janse et al. 2008). In GOTM‐FABM‐PCLake, each fish group is “bound” to each simulated model layer; for instance, piscivorous fish can only predate on fish in their “own” layer. The observed zooplankton community change was likely mediated by an increase in the proportion of piscivorous fish after loading reduction and, with it, lower fish predation on zooplankton (Jeppesen et al. 2005*a*), but this could not be simulated by the model. Nevertheless, the observed fish composition after the model simulation period showed a marked increase in the percentage of piscivores (approximately 5% to 20% from 2002 to 2007; Johansson et al. 2016).

Autocalibration usually involves a search method to determine the most optimal values of selected model parameters that minimize the error between model outputs and observations represented in terms of single‐ or multi‐objective functions or statistics. Many watershed‐runoff models have applied autocalibration, but this has not been a common practice for models with many state variables such as eutrophication or ecosystem models (Rose et al. 2007). This is likely to change as exemplified by a recent study (Luo et al. 2018) presenting simulations of hypoxic events in a polymictic lake done with autocalibration software using a Monte Carlo sampling method for the coupled hydrodynamic‐lake ecosystem model DYRESM‐CAEDYM with 200+ parameter variables. However, the autocalibration was limited to <20 input parameters with a total of 10,000 simulations of temperature and dissolved oxygen. The model presented in this study was calibrated by the autocalibration program ACPy (see footnote 3) for +140 parameters (39% of all parameters available) on multiple state variables depending on the process in focus in the specific calibration step (Table 1). This is the largest set of calibrated parameters derived to date by employing an autocalibration routine for a model in the PCLake family.

The 0D PCLake model has recently been applied to two Danish shallow lakes (manually calibrated), Lake Arreskov and Lake Søbygaard, but with mixed results (Nielsen et al. 2014, Rolighed et al. 2016). Lake Arreskov has demonstrated frequent shifts between a turbid cyanobacteria‐dominated state and a clear‐water, vegetation‐rich state during the past two decades, which a single calibrated parameter set was unable to capture (Nielsen et al. 2014). In contrast, a calibrated PCLake model for shallow Lake Søbygaard simulated seasonal and inter‐annual variations in nutrients and chl *a* concentrations well, but the study did not include a validation period, and the system was less complex than in Lake Arreskov and our study lake, as it did not reach the state where macrophytes occurred (Rolighed et al. 2016). Our calibrated and validated model for Lake Hinge performed better than the above two studies on a daily time step for chl *a* and all nutrient concentrations, except for phosphate.

### Perspectives and recommendations

A clear limitation of model studies with short time series (i.e., 1–2 yr) or aggregate data (e.g., summer means) is how to incorporate the history and trajectory of key indicators of lake water quality, which has proved important with regard to macrophyte dynamics (Sand‐Jensen et al. 2017). A strength of this model study was its long time series of both forcing data (sediment, inflow, and meteorological data) and observational data across several trophic levels, incorporating both loading history (sediment data) and lake ecosystem state change (re‐establishment of macrophytes). The comprehensive data set enabled us to make the model lake‐specific, not just in the forcing and boundary conditions of the model as in some previous PCLake model studies (Janse et al. 2008, Janssen et al. 2017, Li et al. 2019) but also in the calibration of model parameters. As more GOTM‐FABM‐PCLake models are calibrated to specific lakes, it will eventually be possible to consider whether inclusion of depth heterogeneity generally will lead to a more gradual model response to external nutrient load reduction.

Currently, lake managers lack the necessary modeling and decision‐making tools to reliably predict ecosystem state changes and quantify the effectiveness of management measures (Hilt et al. 2017) such as decreased nutrient loading, fish removal, and macrophyte planting. As an example, to reach Danish in‐lake TP criteria (around 0.05 mg TP/L) for good ecological status according to the EU Water Framework Directive (annex V, 2000/60/EC), an empirical model currently used to quantify critical P loads to Danish lakes (Trolle et al. 2015) predicted a required P load reduction of around 50% for Lake Hinge, while the validated and lake‐specific GOTM‐FABM‐PCLake model predicted a P load reduction of around 20–30% for an ecosystem state change to occur. The differences in predictions can have great economic consequences once management plans are to be implemented in practice, not least in agricultural‐dominated catchments like Lake Hinge.

To improve the prediction of macrophyte dynamics and reduce the sensitivity of bifurcation analysis, we propose that more shallow lake model studies focus on lakes with a history of macrophyte establishment or die‐back to explore the range of different macrophyte parameterizations and potentially improve the conceptual design of the model. We hypothesize that the models will likely need to include more functional groups of macrophytes to encompass different macrophyte communities at different trophic states. We also highlight the need for including at least two user‐defined groups of zooplankton in temperate shallow lakes, for example a copepod and a cladoceran group, to encompass the seasonal dynamics of zooplankton biomass and composition as well as changes in the seasonal grazing pressure on phytoplankton. With refinements of the descriptions of the fish, macrophyte, and zooplankton modules in FABM‐PCLake that are already underway, GOTM‐FABM‐PCLake will offer an increasingly powerful tool for managers to evaluate the effectiveness of management measures and to estimate the required nutrient loading reductions to reach good lake ecosystem quality.

## Supporting information

Appendix S1

Metadata S1
