# Supplementary material for: Predicting ecosystem state changes in shallow lakes using an aquatic ecosystem model: Lake Hinge, Denmark, an example
Source: Ecol Appl. 2020 Jun 11;30(7):e02160. doi: 10.1002/eap.2160 (PMC7583379; doi:10.1002/eap.2160)
Supplement: Supplementary file 1 — Appendix S1 [file EAP-30-e02160-s001.pdf]

Supporting Information. Andersen, T.K., A. Nielsen, E. Jeppesen, F. Hu, K. Bolding, Z. Liu, M. Søndergaard, L.S. Johansson, and D. Trolle. 2020. Predicting ecosystem state changes in shallow lakes using an aquatic ecosystem model: Lake Hinge, Denmark, an example. Ecological Applications.

Appendix S1

Table S1: Parameter values for calibrated FABM-PCLake model for Lake Hinge, Denmark

* All parameter values are included in the *fabm.yaml* file in the Lake Hinge model setup folder (Data S2)

ID	Parameter	Module	Definition	Unit	Parameter value			
					Assigned	*Default*	*Literature*	ref
2	**cExtSpPOM**	abiotic_water	specific extinction factor for POM	m^2/gDW	**0.1**	0.15		
4	**cKPAdsOx**	abiotic_water	P adsorption affinity under oxidized conditions	m^3/gP	**1.407**	0.6		
6	**cRelPAdsD**	abiotic_water	maximum P adsorption per g DW	gP/gD	**7.8E-05**	3E-05		
7	**cRelPAdsFe**	abiotic_water	maximum P adsorption per g Fe	gP/gFe	**0.089**	0.065		
8	**cThetaAer**	abiotic_water	temperature coefficient for reaeration	1/e^oC	**1.017**	1.024		
9	**cThetaNitr**	abiotic_water	temperature coefficient for nitrification	[-]	**1.03**	1.08		
10	**cVSetPOM**	abiotic_water	maximum settling rate of POM	m/day	**-0.28**	-0.25		
11	**cVSetIM**	abiotic_water	maximum settling rate of inorganic matter	m/day	**-1.045**	-1		
13	**fFeDIM**	abiotic_water	Fe content of inorganic matter	gFe/gD	**0.042**	0.01		
14	**fRedMax**	abiotic_water	maximum reduction factor of P adsorption affinity	[-]	**0.911**	0.9		
15	**hNO3Denit**	abiotic_water	quadratic half saturation NO3 conc. for denitrification	mgN/L	**1.217**	2		
16	**hO2BOD**	abiotic_water	half saturation oxygen conc. for BOD	mgO2/L	**1.395**	1		
17	**hO2Nitr**	abiotic_water	half saturation oxygen conc. for nitrification	mgO2/L	**2.037**	2		
18	**kNitrW**	abiotic_water	nitrification rate constant in water	d^{-1}	**0.198**	0.1		
19	**kPSorp**	abiotic_water	P adsorption rate constant	d^{-1}	**0.408**	0.05		
20	**NO3PerC**	abiotic_water	denitrified NO3 per mol C mineralized	molNO3	**0.853**	0.8		
21	**O2PerNH4**	abiotic_water	used O2 per mol NH4+ nitrified	molO2	**2.067**	2		
22	**cThetaMinPOMW**	abiotic_water	temperature coefficient for mineralization from POM to DOM	[-]	**1.076**	1.07		

23	**kDMinPOMW**	abiotic_water	decomposition constant for POM-DW to DOM-DW	d^{-1}	**0.001**	0.01		
24	**kNMinPOMW**	abiotic_water	decomposition constant for POM-N to DOM-N	d^{-1}	**0.008**	0.01		
25	**kPMinPOMW**	abiotic_water	decomposition constant for POM-P to DOM-P	d^{-1}	**0.012**	0.01		
27	**cThetaMinDOMW**	abiotic_water	temperature coefficient for DOM mineralization	[-]	**1.073**	1.07		
28	**kDMinDOMW**	abiotic_water	mineralization constant of dissolved organic DW	d^{-1}	**0.054**	0.01		
29	**kNMinDOMW**	abiotic_water	mineralization constant of dissolved organic N	d^{-1}	**0.008**	0.01		
30	**kPMinDOMW**	abiotic_water	mineralization constant of dissolved organic P	d^{-1}	**0.011**	0.01		
35	**O2PerNH4**	abiotic_sediment	O2 used per mol NH4 nitrified	mol	**2.032**	2		
36	**kNitrS**	abiotic_sediment	nitrification rate constant	d^{-1}	**1.237**	1		
37	**cThetaNitr**	abiotic_sediment	temperature coefficient for nitrification	[-]	**1.06**	1.08		
39	**hNO3Denit**	abiotic_sediment	quadratic half-sat. NO3 conc. for denitrification	mgN/l	**1.291**	2		
40	**kPSorp**	abiotic_sediment	P sorption rate constant	d^{-1}	**0.041**	0.05		
41	**cRelPAdsD**	abiotic_sediment	max. P adsorption per g DW	gP/gD	**3.2E-05**	3E-05		
42	**cRelPAdsFe**	abiotic_sediment	max. P adsorption per g Fe	gP/gFe	**0.062**	0.065	max 0.067, 0.1	i, j
43	**fFeDIM**	abiotic_sediment	Fe content of inorg. matter	gFe/gD	**0.054**	0.01		x
46	**fRedMax**	abiotic_sediment	max. reduction factor of P adsorption affinity	[-]	**0.953**	0.9		
47	**cKPAdsOx**	abiotic_sediment	P adsorption affinity under oxidized conditions	m^3/gP	**0.752**	0.6		
49	**coPO4Max**	abiotic_sediment	max. SRP conc. in pore water	mgP/L	**1.742**	1	0.0-6.1	f
50	**bPorS**	abiotic_sediment	sediment porosity	m3 water /m^3 sediment	**0.85**	0.847947		
51	**cThetaDif**	abiotic_sediment	temperature coefficient for diffusion	[-]	**1.031**	1.02		
54	**cTurbDifNut**	abiotic_sediment	bioturbation factor for diffusion	[-]	**4.234**	5		
55	**bPorCorS**	abiotic_sediment	sediment porosity	m3 water/m^3 sediment	**0.737**	0.737275		
58	**kO2Dif**	abiotic_sediment	molecular O2 diffusion constant	m^2/d	**0.000208**	2.6E-05		
59	**cTurbDifO2**	abiotic_sediment	bioturbation factor for diffusion	[-]	**6.56**	5		
61	**cThetaMinPOMS**	abiotic_sediment	temperature coeff. for sediment mineralization of POM to DOM	[-]	**1.097**	1.07		
62	**kDMinPOMS**	abiotic_sediment	mineralization constant in sediment from POM-DW to DOM-DW	d^{-1}	**0.003**	0.002		
64	**kPMinPOMS**	abiotic_sediment	mineralization constant in sediment from POM-P to DOM-P	d^{-1}	**0.004**	0.002		
66	**cThetaMinDOMS**	abiotic_sediment	exp. temperature constant of sediment mineralization	[-]	**1.136**	1.07		

67	**kDMinDOMS**	abiotic_sediment	mineralization constant for sediment dissolved organic matter	d^{-1}	**0.001**	0.002		
68	**kNMinDOMS**	abiotic_sediment	mineralization constant for sediment dissolved organic N	d^{-1}	**0.001**	0.002		
71	**kDDifDOM**	abiotic_sediment	molecular diffusion constant for dissolved organic matter	m^2/d	**2.7E-05**	0.000112		
72	**kNDifDOM**	abiotic_sediment	molecular diffusion constant for dissolved organic N	m^2/d	**5.3E-05**	0.000112		
73	**kPDifDOM**	abiotic_sediment	molecular diffusion constant for dissolved organic P	m^2/d	**0.000207**	0.000112		
74	**kSiDifDOM**	abiotic_sediment	molecular diffusion constant for dissolved organic Si	m^2/d	**0**	0.000112		
77	**cSigTmBlue**	phytoplankton_water	temperature constant blue-greens (sigma in Gaussian curve)	degree C	**11.029**	12		
78	**cTmOptBlue**	phytoplankton_water	optimum temperature of blue-greens	degree C	**25.908**	25		
81	**cPDDiatMin**	phytoplankton_water	minimum P/DW ratio for diatoms	mgP/mgDW	**0.002**	0.0005	0.002	d
82	**cPDDiatMax**	phytoplankton_water	maximum P/DW ratio for diatoms	mgP/mgDW	**0.011**	0.005	0.02	d
83	**cNDDiatMin**	phytoplankton_water	minimum N/DW ratio for diatoms	mgN/mgDW	**0.011**	0.01		
84	**cNDDiatMax**	phytoplankton_water	maximum N/DW ratio for diatoms	mgN/mgDW	**0.08**	0.05		
85	**hSiAssDiat**	phytoplankton_water	half-saturation constant for Si influence on diatoms	mgSi/L	**0.01**	0.09		
86	**cPDGrenMin**	phytoplankton_water	minimum P/DW ratio greens	mgP/mgDW	**0.002**	0.0015		
91	**cPDBlueMin**	phytoplankton_water	minimum P/DW ratio blue-greens	mgP/mgDW	**0.003**	0.0025	0.002	d
93	**cNDBlueMin**	phytoplankton_water	minimum N/DW ratio blue-greens	mgN/mgDW	**0.035**	0.03	0.09	d
94	**cNDBlueMax**	phytoplankton_water	maximum N/DW ratio blue-greens	mgN/mgDW	**0.16**	0.15	0.01	d
99	**cLOptRefDiat**	phytoplankton_water	optimum PAR for diatoms at 20 degrees C	W/m^2	**29.341**	54	2-27	a
100	**cLOptRefGren**	phytoplankton_water	optimum PAR for greens at 20 degrees C	W/m^2	**56.151**	30	4-35	a
101	**cLOptRefBlue**	phytoplankton_water	optimum PAR for blue-greens at 20 degrees C	W/m^2	**51.881**	13.6	36 (4-69)	a
102	**cMuMaxBlue**	phytoplankton_water	maximum growth rate blue-greens	d^{-1}	**1.068**	0.6	1.1 (0.5-1.7)	b, e
103	**cMuMaxGren**	phytoplankton_water	maximum growth rate greens	d^{-1}	**0.98**	1.5	0.2-2.9	c
104	**cMuMaxDiat**	phytoplankton_water	maximum growth rate diatoms	d^{-1}	**1.987**	2	1.5 (0.3-2.7)	e
111	**cVPUptMaxDiat**	phytoplankton_water	maximum P uptake capacity of diatoms	$mgP\ mgDW^{-1}\ d^{-1}$	**0.012**	0.01	0.02-0.5	e
113	**cVPUptMaxBlue**	phytoplankton_water	maximum P uptake capacity of blue-greens	$mgP\ mgDW^{-1}\ d^{-1}$	**0.037**	0.04	0.02-0.5	e
114	**cAffPUptDiat**	phytoplankton_water	initial P uptake affinity diatoms	$L\ mgDW^{-1}\ d^{-1}$	**0.143**	0.2		
116	**cAffPUptBlue**	phytoplankton_water	initial P uptake affinity blue-greens	$L\ mgDW^{-1}\ d^{-1}$	**0.745**	0.8		
117	**cVNUptMaxDiat**	phytoplankton_water	maximum N uptake capacity of diatoms	$mgN\ mgDW^{-1}\ d^{-1}$	**0.103**	0.07		
119	**cVNUptMaxBlue**	phytoplankton_water	maximum N uptake capacity of blue-greens	$mgN\ mgDW^{-1}\ d^{-1}$	**0.077**	0.07		
120	**cAffNUptDiat**	phytoplankton_water	initial N uptake affinity diatoms	$L\ mgDW^{-1}\ d^{-1}$	**0.428**	0.2		

122	**cAffNUptBlue**	phytoplankton_water	initial N uptake affinity bluegreens	L $mgDW^{-1}$ d^{-1}	**0.561**	0.2		
123	**fDissMortPhyt**	phytoplankton_water	soluble nutrient fraction of dead algae	[-]	**0**	0.2		
127	**cChDDiatMin**	phytoplankton_water	minimum chlorophyll/C ratio for diatoms	mgChl/mgDW	**0.018**	0.004	0.005-0.016	c
128	**cChDDiatMax**	phytoplankton_water	maximum chlorophyll/C ratio for diatoms	mgChl/mgDW	**0.025**	0.012	0.005-0.016	c
129	**cChDGrenMin**	phytoplankton_water	minimum chlorophyll/C ratio for greens	mgChl/mgDW	**0.02**	0.01	0.01-0.02	c
130	**cChDGrenMax**	phytoplankton_water	maximum chlorophyll/C ratio for greens	mgChl/mgDW	**0.025**	0.02	0.01-0.02	c
131	**cChDBlueMin**	phytoplankton_water	minimum chlorophyll/C ratio for blue-greens	mgChl/mgDW	**0.04**	0.005	0.016-0.09	c
132	**cChDBlueMax**	phytoplankton_water	maximum chlorophyll/C ratio for blue-greens	mgChl/mgDW	**0.05**	0.015	0.016-0.09	c
135	**cVSetBlue**	phytoplankton_water	settling rate of blue-greens	m/d	**0.044**	0.06		
136	**cExtSpDiat**	phytoplankton_water	specific extinction of diatoms	m^2/gDW	**0.2**	0.25		
137	**cExtSpGren**	phytoplankton_water	specific extinction of greens	m^2/gDW	**0.2**	0.25		
139	**UseLightMethodGren**	phytoplankton_water	light method	[-]	**2**	1		
142	**cDBlueMinW**	phytoplankton_water	minimum blue-green algae concentration in system	gDW/m^3	**0.0001**	1E-05		
143	**cDGrenMinW**	phytoplankton_water	minimum green algae concentration in system	gDW/m^3	**0.0001**	1E-05		
144	**cDDiatMinW**	phytoplankton_water	minimum diatom concentration in system	gDW/m^3	**0.0001**	1E-05		
145	**fPrimDOMW**	phytoplankton_water	fraction of dissolved organic matter from water column phytoplankton	[-]	**0.263**	0.5		
152	**kMortDiatS**	phytoplankton_sediment	mortality rate of settled diatoms	d^{-1}	**0.085**	0.05		
155	**cPDDiatMin**	phytoplankton_sediment	minimum P/DW ratio diatoms	mgP/mgDW	**0.001**	0.0005		
159	**cPDGrenMin**	phytoplankton_sediment	minimum P/DW ratio greens	mgP/mgDW	**0.002**	0.0015		
163	**cPDBlueMin**	phytoplankton_sediment	minimum P/DW ratio blue-greens	mgP/mgDW	**0.003**	0.0025		
164	**fDissMortPhyt**	phytoplankton_sediment	soluble nutrient fraction of dead algae	[-]	**0.01**	0.2		
175	**fPrimDOMS**	phytoplankton_sediment	fraction of DOM from settled phytoplankton	[-]	**0.25**	0.5		
180	**cPDVegMin**	macrophytes	minimum P/DW ratio macrophytes	mgP/mg	**0.001**	0.0008		
181	**cPDVegMax**	macrophytes	maximum P/DW ratio macrophytes	mgP/mgD	**0.004**	0.0035		
182	**cMuMaxVeg**	macrophytes	maximum growth rate of macrophytes at 20 degrees C	d^{-1}	**0.09**	0.2	0.05-0.5	g
183	**cDCarrVeg**	macrophytes	maximum macrophyte standing crop	gDW/m^2	**100**	400		
194	**hLRefVeg**	macrophytes	half-saturation for influence of light on macrophytes	W/m^2PAR	**10.5**	17	9-22, 3-11	m, n

208	**bPorS**	macrophytes	sediment porosity	[-]	**0.85**	0.847947		
213	**cCPerDW**	macrophytes	C content of organic matter	gC/gDW	**0.45**	0.4		
216	**cHeightVeg**	macrophytes	macrophytes height	m	**0.2**	1		
217	**cExtSpVeg**	macrophytes	specific extinction of macrophytes	m^2/gDW	**0.001**	0.01		
218	**cDVegMin**	macrophytes	minimum dry weight of macrophytes in system	gDW/m^2	**0.0001**	1E-05		
221	**cSigTmZoo**	zooplankton	temperature constant for zooplankton	degree C	**11.06**	13		
222	**cTmOptZoo**	zooplankton	optimum temperature for zooplankton	degree C	**25.453**	25		
226	**cPrefDiat**	zooplankton	selection factor for diatoms	[-]	**0.9**	0.75		
228	**cPrefBlue**	zooplankton	selection factor for blue-greens	[-]	**0.342**	0.125		
229	**cPrefPOM**	zooplankton	selection factor for particulate organic matter	[-]	**0.191**	0.25		
230	**hFilt**	zooplankton	half-saturation constant for food conc. on zooplankton	gDW/m^3	**0.801**	1	0.5 - 3.0	e
231	**fDAssZoo**	zooplankton	dry weight assimilation efficiency of zooplankton	[-]	**0.301**	0.35	0 - 1	e
232	**cFiltMax**	zooplankton	maximum filtering rate	L $mgDW^{-1}$ d^{-1}	**4.573**	4.5	0.2 - 6.5	e
235	**fDissEgesZoo**	zooplankton	inorganic soluble nutrient fraction of zooplankton egested food	[-]	**0.01**	0.25		
236	**fDissMortZoo**	zooplankton	inorganic soluble nutrient fraction of dead zooplankton	[-]	**0.01**	0.1		
238	**fZooDOMW**	zooplankton	dissolved organic fraction from zooplankton	[-]	**0.35**	0.5		
256	**hDZooFiJv**	fish	half-saturation zooplankton for zooplanktivorous fish predation	g/m^2	**1.204**	1.25		
259	**kMortFiJv**	fish	specific mortality of zooplanktivorous fish	d^{-1}	**0.001**	0.00137		
266	**cCovVegMin**	fish	minimum submerged macrophyte coverage for piscivorous fish	%	**1.79**	40		
269	**kDAssPisc**	fish	maximum assimilation rate for piscivorous fish	d^{-1}	**0.078**	0.025		
270	**hDVegPisc**	fish	half-saturation constant for macrophytes on piscivorous fish	g/m^2	**0.014**	5		
276	**fDissMortPisc**	fish	soluble nutrient fraction of dead piscivorous fish	[-]	**0.01**	0.1		
283	**cDFiJvMin**	fish	minimum zooplanktivorous fish biomass in system	gDW/m^2	**1E-05**	0.0001		
284	**cDFiAdMin**	fish	minimum benthivorous fish biomass in system	gDW/m^2	**1E-05**	0.0001		
285	**cDPiscMin**	fish	minimum piscivorous fish biomass in system	gDW/m^2	**1E-05**	0.0001		
286	**fFisDOMW**	fish	dissolved organic matter fraction from fish	[-]	**0**	0.5		
290	**hDBentFiAd**	fish	half-saturation constant for zoobenthos on adult fish	g/m^2	**1.576**	2.5		
307	**cPDDiatMin**	zoobenthos	minimum P/DW ratio diatoms	mgP/mgDW	**0.001**	0.0005		
309	**cPDGrenMin**	zoobenthos	minimum P/DW ratio greens	mgP/mgDW	**0.002**	0.0015		
311	**cPDBlueMin**	zoobenthos	minimum P/DW ratio blue-greens	mgP/mgDW	**0.003**	0.0025		

319	**fBenDOMS**	zoobenthos	dissolved organic fraction from zoobenthos	[-]	**0.03**	0.5		
323	**kTurbFish**	auxiliary	relative resuspension by adult fish browsing	g $gfish^{-1}$ d^{-1}	**4.803**	1	max 5	k
333	**bPorS**	auxiliary	sediment porosity	m3water/m3sediment	**0.85**	0.847947		
337	**cVSedPOM**	auxiliary	maximum sedimentation velocity of POM	m/d	**0.1**	0.25		
338	**cVSedDiat**	auxiliary	sedimentation velocity of diatoms	m/d	**0.557**	0.5		
340	**cVSedBlue**	auxiliary	sedimentation velocity of blue-greens	m/d	**0.35**	0.06		
350	**cPDDiatMin**	auxiliary	minimum P/DW ratio for diatoms	mgP/mgDW	**0.001**	0.0005		
352	**cPDGrenMin**	auxiliary	minimum P/DW ratio for greens	mgP/mgDW	**0.002**	0.0015		
354	**cPDBlueMin**	auxiliary	minimum P/DW ratio for blue-greens	mgP/mgDW	**0.003**	0.0025		
361	**crt_shear**	auxiliary	critical shear stress	N/m	**0.051**	0.005	0.001-0.02 , 0.01-1	l, o
363	**alpha**	auxiliary	gross rate of sediment erosion	g m^{-2} d^{-1}	**3004.217**	9000	550-11000	p
365	**cVSedMain**	auxiliary	depth averaged sedimentation velocity	m d^{-1}	**0.993**	0.5		

x Estimated from field data from Danish lakes

a O'Sullivan & Reynolds, 2004

c Reynolds, 2006

d Kalff, 2002, p. 328

e Bowie et al., 1985

f Eckerrot & Petterson, 1993

g Janse, 2005

h Leidy & Ploskey, 1980

i Caraco, Cole, & Likens, 1993

j Jensen, Kristensen, Jeppesen, & Skytthe, 1992

k Breukelaar *et al.*, 1994

l Trolle, Hamilton, Pilditch, Duggan, & Jeppesen, 2011

m Sand-jensen, Binzer, Middelboe, Binzer, & Middelboe, 2007

n van Nes, Scheffer, van den Berg, & Coops, 2003

o Hipsey, Antenucci, Romero, & Hamilton, 2010

p Hamilton & Mitchell, 1996

References

Bowie, G. L., W. B. Mills, D. B. Porcella, C. L. Campbell, J. R. Pagenkopf, G. L. Rupp, K. M. Johnson, P. W. H. Chan, S. A. Gherini, and C. E. Chamberlin. 1985. Rates, Constants, and Kinetics Formulations in Surface Water Quality Modeling (Second Edition). Enviromental research laboratory office of research and development U.S. enviromental protection agency. Athens, Georgia.

Breukelaar, A. W., E. H. R. R. Lammens, J. G. P. K. Breteler, and I. Tatrai. 1994. Effects of benthivorous bream (Abramis brama) and carp (Cyprinus carpio) on sediment resuspension and concentrations of nutrients and chlorophyll a. Freshwater Biology 32:113–121.

Caraco, N. F., J. J. Cole, and G. E. Likens. 1993. Sulfate control of phosphorus availability in lakes. Hydrobiologia 253:275–280.

Eckerrot, Å., and K. Petterson. 1993. Pore water phosphorus and iron concentrations in a shallow, eutrophic lake - indications of bacterial regulation. Hydrobiologia 253:165–177.

Hamilton, D. P., and S. F. Mitchell. 1996. An empirical model for sediment resuspension in shallow lakes. Hydrobiologia 317:209–220.

Hipsey, M. R., J. P. Antenucci, J. R. Romero, and D. P. Hamilton. 2010. Computational Aquatic Ecosystem Dynamics Model: CAEDYM science manual.

Janse, J. H. 2005. Model studies on the eutrophication of shallow lakes and ditches.

Jensen, H. S., P. Kristensen, E. Jeppesen, and A. Skytthe. 1992. Iron:phosphorus ratio in surface sediment as an indicator of phosphate release from aerobic sediments in shallow lakes. Hydrobiologia 235–236:731–743.

Kalff, J. 2002. Limnology. Prentice Hall.

Leidy, G. R., and G. R. Ploskey. 1980. Simulation modeling of zooplankton and benthos in resevoirs: Documentation and development of model constructs.

van Nes, E. H., M. Scheffer, M. S. van den Berg, and H. Coops. 2003. Charisma: a spatial explicit simulation model of submerged macrophytes. Ecological Modelling 159:103–116.

O'Sullivan, P. E. (Patrick E. ., and C. S. Reynolds. 2004. The lakes handbook. Volume 1, Limnology and limnetic ecology. Blackwell Science.

Reynolds, C. S. 2006. Ecology of phytoplankton. Cambridge University Press.

Sand-Jensen, A. K., T. Binzer, A. L. Middelboe, K. S. T. Binzer, and A. L. Middelboe. 2007. Scaling of Photosynthetic Production of Aquatic Macrophytes: A Review. Oikos 116:280–294.

Trolle, D., D. P. Hamilton, C. A. Pilditch, I. C. Duggan, and E. Jeppesen. 2011. Predicting the effects of climate change on trophic status of three morphologically varying lakes: Implications for lake restoration and management. Environmental Modelling and Software 26:354–370.
