# Supplementary material for: Predicting ecosystem state changes in shallow lakes using an aquatic ecosystem model: Lake Hinge, Denmark, an example
Source: Ecol Appl. 2020 Jun 11;30(7):e02160. doi: 10.1002/eap.2160 (PMC7583379; doi:10.1002/eap.2160)
Supplement: Supplementary file 2 — Metadata S1 [file EAP-30-e02160-s002.pdf]

Andersen, T.K., A. Nielsen, E. Jeppesen, F. Hu, K. Bolding, Z. Liu, M. Søndergaard, L.S. Johansson, and D. Trolle. 2020. Predicting ecosystem state changes in shallow lakes using an aquatic ecosystem model: Lake Hinge, Denmark, an example. Ecological Applications.

Data S1

Data and model configuration files to run GOTM-FABM-PCLake model

---

Authors

Tobias Kuhlmann Andersen
Department of Bioscience, Aarhus University,
Vejlsøvej 25, 8600 Silkeborg, Denmark
tka@bios.au.dk

Anders Nielsen
Department of Bioscience, Aarhus University,
Vejlsøvej 25, 8600 Silkeborg, Denmark
an@bios.au.dk

Karsten Bolding
Department of Bioscience, Aarhus University,
Vejlsøvej 25, 8600 Silkeborg, Denmark
bolding@bios.au.dk

Liselotte S. Johansson
Department of Bioscience, Aarhus University,
Vejlsøvej 25, 8600 Silkeborg, Denmark
lsj@bios.au.dk

Dennis Trolle
Department of Bioscience, Aarhus University,
Vejlsøvej 25, 8600 Silkeborg, Denmark
trolle@bios.au.dk

File list (files found within DataS1.zip)

Folder: lake_hinge_model_setup

```
hypsograph.dat
inflow.dat
inflow_chem_adj.dat
meteo_file.dat
outflow.dat
airsea.nml
fabm_input.nml
gotm_fabm.nml
gotmmean.nml
gotmrun.nml
gotmturb.nml
manipulations.nml
obs.nml
streams.nml
fabm.yaml
fabm_default.yaml
output.yaml
```

Subfolder: observations

```
BLUE.obs
CHLA.obs
DIAT.obs
DO.obs
GREN.obs
IM.obs
LOI.obs
NH4.obs
NO23.obs
PO4.obs
TEMPV.obs
TOTN.obs
TOTP.obs
ZOO.obs
```

Subfolder: scenarios

Subfolders: eutrophication and oligotrophication

```
inflow.dat
meteo_file.dat
outflow.dat
inflow_chem_PX_N100.dat*
```

***X** in `inflow_chem_PX_N100.dat` refers to percentage loading of baseline in scenario simulations (see below for further details)

Description

Lake Hinge model configuration files

`hypsograph.dat` - hypsograph for Lake Hinge

`inflow.dat` – time series with estimated monthly discharge to Lake Hinge

`inflow_chem_adj.dat` – time series with estimated monthly nutrient concentrations in Lake Hinge

`meteo_file.dat` – time series with weather forcing for Lake Hinge from ECMWF dataset

`outflow.dat` - time series with estimated monthly discharge out of Lake Hinge

`airsea.nml` - contains information on heat, momentum and freshwater fluxes between the ocean and the atmosphere as well as the incoming solar radiation fluxes.

`fabm_input.nml`- file points to input files for nutrient inflow to Lake Hinge

`gotm_fabm.nml`- file coupling GOTM to FABM-PCLake

`gotmmean.nml`- contains information on water layers in the 1D model, e.g. number of layers and layer height

`gotmrun.nml`- contains mainly parameters concerning the model run, the time step, the model time, the output format, etc.

`gotmturb.nml`- information about the applied turbulence models in GOTM.

`manipulations.nml`- contains time steps and percentage reductions in fish biomass during ice winters.

`obs.nml`- file contains information about "observed"quantities that are used to either force the model (like internal and external pressure gradients) or for comparison with computed results.

`streams.nml` - information on how to read observed inflow and outflow profiles and times series.

`output.yaml`- file describes how to output model simulation

`fabm.yaml` - parameter values for the calibrated FABM-PCLake model

`fabm_default.yaml` - default parameter values for the FABM-PCLake model

`gotm.exe` - executable of aquatic ecosystem model complex GOTM-FABM-PCLake used in lake model study of Lake Hinge. For code and model description, please see:

Hu, F., K. Bolding, J. Bruggeman, E. Jeppesen, M. R. Flindt, L. Van Gerven, J. H. Janse, A. B. G. Janssen, J. J. Kuiper, W. M. Mooij, and D. Trolle. 2016. FABM-PCLake -

Linking aquatic ecology with hydrodynamics. Geoscientific Model Development 9:2271–2278.

Observation files

Time series with observed variables in Lake Hinge from 1990-2007:

`BLUE.obs` - cyanobacteria biomass concentrations (mg DW/L)

`CHLA.obs` - total chlorophyll *a* concentrations (µg/L)

`DIAT.obs` - diatom biomass concentrations (mg DW/L)

`DO.obs` - dissolved oxygen concentrations (mg DO/L)

`GREN.obs` - 'other algae' biomass concentrations (mg DW/L)

`IM.obs` - inorganic matter concentrations (mg/L)

`LOI.obs` - particulate organic matter concentrations (mg/L)

`NH4.obs` - ammonium concentrations in Lake Hinge concentrations (mg/L)

`NO23.obs` - nitrate and nitrite concentrations (mg/L)

`PO4.obs` - phosphate concentrations (mg/L)

`TEMPV.obs` - temperature concentrations (mg/L)

`TOTN.obs` - total nitrogen concentrations (mg/L)

`TOTP.obs` - total phosphorous concentrations (mg/L)

`ZOO.obs` - zooplankton biomass concentrations (mg DW/L)

Scenarios files

`inflow.dat`, `inflow_chem.dat`, `meteo_file.dat` and `outflow.dat` files to run both eutrophication and oligotrophication scenarios described in this study. Numbers in file names for `inflow_chem_PX_N100.dat` refers to the percentage P and N loading, respectively, of baseline, i.e. `inflow_chem-P10.0-N100.dat` contains time series of inflow nutrient concentrations with 10% phosphorous and 100% nitrogen loading of the baseline.
